# Revisiting enteric methane emissions from domestic ruminants and their δ^13^C_CH4_ source signature

**DOI:** 10.1038/s41467-019-11066-3

**Published:** 2019-07-31

**Authors:** Jinfeng Chang, Shushi Peng, Philippe Ciais, Marielle Saunois, Shree R. S. Dangal, Mario Herrero, Petr Havlík, Hanqin Tian, Philippe Bousquet

**Affiliations:** 1 Laboratoire des Sciences du Climat et de l’Environnement, CEA-CNRS-UVSQ/IPSL, Université Paris Saclay, 91191 Gif sur Yvette, France; 2 Sino-French Institute for Earth System Science, College of Urban and Environmental Sciences, Peking University, Beijing, 100871 China; 3 International Center for Climate and Global Change Research and School of Forestry and Wildlife Sciences, Auburn University, Auburn, AL 36849 USA; 4 Woods Hole Research Center, Falmouth, MA 02540 USA; 5 Commonwealth Scientific and Industrial Research Organization, St Lucia, QLD 4067 Australia; 6 International Institute for Applied Systems Analysis, A-2361 Laxenburg, Austria; 7 Present Address: International Institute for Applied Systems Analysis, A-2361 Laxenburg, Austria

**Keywords:** Stable isotope analysis, Carbon cycle, Atmospheric chemistry, Atmospheric chemistry

## Abstract

Accurate knowledge of ^13^C isotopic signature (δ^13^C) of methane from each source is crucial for separating biogenic, fossil fuel and pyrogenic emissions in bottom-up and top-down methane budget. Livestock production is the largest anthropogenic source in the global methane budget, mostly from enteric fermentation of domestic ruminants. However, the global average, geographical distribution and temporal variations of the δ^13^C of enteric emissions are not well understood yet. Here, we provide a new estimation of C3-C4 diet composition of domestic ruminants (cattle, buffaloes, goats and sheep), a revised estimation of yearly enteric CH_4_ emissions, and a new estimation for the evolution of its δ^13^C during the period 1961–2012. Compared to previous estimates, our results suggest a larger contribution of ruminants’ enteric emissions to the increasing trend in global methane emissions between 2000 and 2012, and also a larger contribution to the observed decrease in the δ^13^C of atmospheric methane.

## Abstract (web summary)

Global average, geographical distribution and temporal variations of the 13C isotopic signature of enteric fermentation emissions are not well understood. Here the authors established a global dataset and show a larger emission increase between the two periods (2002–2006 and 2008–2012) than previous studies.

## Introduction

Methane has important anthropogenic emissions, and is the second largest driver of global radiative forcing (0.97 ± 0.16 W m^−2^) after CO_2_^1^. Understanding the global methane budget and its sources is crucial for climate mitigation efforts. Both process-based (bottom-up) and atmospheric-based (top-down) methods are used to constrain the sources and sinks of methane. However, large uncertainties exist in both approaches, which limits the complete understanding of the global methane budget.

Measurements of atmospheric methane concentrations, including their trend and gradients between stations of the surface in situ network, together with a priori spatial and temporal patterns of source type information are used by atmospheric inversion systems to produce optimized estimates of broad source categories (the Global Carbon Project^2^) and of the global budget, including surface sources and atmospheric sinks. The measurements of the ^13^C stable isotope composition of atmospheric methane (i.e., δ^13^C_CH4-atm_) bring additional constraints for attributing methane emission sources^3–6^. The ^13^C/^12^C-ratio in atmospheric CH_4_ (δ^13^C_CH4-atm_; expressed in δ-notation relative to Vienna Pee Dee Belemnite (VPDB)-standard) is controlled by the relative contributions from different source types with distinctive isotope signatures, δ^13^C_CH4-sources_, and by the isotopic fractionation during reaction with atmospheric OH and chlorine radicals. Reference ^6^ and further ref. ^7^ revised the δ^13^C of methane sources and concluded that microbial sources, including wetlands, rice paddies, ruminant enteric fermentation and waste emissions, have a mean δ^13^C_CH4_ of ~ −61.7 ± 6.2‰, fossil-fuel sources have a mean δ^13^C_CH4_ of ~ −44.8 ± 10.7‰, and pyrogenic sources from biomass burning have a mean δ^13^C_CH4_ of ~−26.2 ± 4.8‰. The uncertainty and variability (in space and time) of these signatures directly affects the accuracy of source attribution by inversions. For example, the observed plateau of atmospheric methane concentration during 1999–2006, the renewed concentration-rise after 2006, and associated δ^13^C_CH4-atm_ changes were used to quantify the role of different sources^5^. However, biases in the mean isotopic signatures of individual sources and how they change with time translate into potentially large uncertainties on the inferred trends of emission in this approach^8^.

Livestock production is the largest anthropogenic source in the global methane budget (103 [95–109] Tg CH_4_ yr^−1^ during 2000–2009^2^). Enteric fermentation from ruminants dominates this source and accounts for emission of 87–97 Tg CH_4_ yr^−1^ during 2000–2009^9–12^. Livestock manure management has a smaller contribution. Cattle, buffaloes, goats, and sheep are the main ruminant livestock types emitting CH_4_ and altogether represent 96% of the global enteric fermentation source^9^. Several methodologies are recommended by IPCC^13^ (Vol. 4, Chapter 10.3) to estimate national to global enteric methane emissions. The Tier 1 method that uses the livestock population data and default emission factors, the Tier 2 approach uses a more detailed country-specific data on gross energy intake and methane conversion factors for specific livestock categories, and the Tier 3 approach allows detailed parameterization of rumen fermentation. Given the large magnitude of ruminant emissions (F_CH4-ruminant_), its δ^13^C (δ^13^C_CH4-ruminant_) needs to be assessed as precisely as possible regionally for constraining the global mix of emissions using inversion models driven by atmospheric CH_4_ and isotope data.

Photosynthesis pathways differentiate C3 and C4 plants^14^. C4 plants contain more ^13^C than C3 plants relative to ^12^C. This difference in isotopic ratio causes methane emissions from ruminants with a higher C4 diet to be isotopically heavier (less negative δ^13^C_CH4_) than those with a C3 diet. Therefore, to assess the δ^13^C_CH4-ruminant_, it is critical to first differentiate the C3 vs. C4 feed composition in ruminant diet. To our knowledge, the proportions of C3 vs. C4 crops fed to ruminants (as concentrate feeds), as opposed to pig and poultry and their temporal changes, have not been investigated at national and global scale, since FAOSTAT only provides total feed crops for all livestock types grouped together. In addition, given the fact that C4 photosynthetic pathway predominates in warm season/low precipitation grass species, the strong increase in livestock number in tropical regions, such as South America, Africa, and South and Southeast Asia, should also increase the value of δ^13^C_CH4-ruminant_ (i.e., heavier). Few studies have considered the impacts of shifting C4–C3 diet composition in ^13^C constraints on the global CH_4_ budget. Reference ^6^ estimated a global weighted mean δ^13^C_CH4-ruminant_ value of −66.8 ± 2.8‰ using an observation-based C4–C3 diet fraction of the United States only and made assumptions for the rest of the world. The spatial distribution and temporal trends of livestock δ^13^C_CH4-ruminant_ is thus a research gap.

In addition, atmospheric isotope signatures of CO_2_ (δ^13^C_CO2-atm_) decreased by −1.3‰ from 1960 to 2012 (see observations from the Scripps CO_2_ Program; http://scrippsco2.ucsd.edu/; data compiled in ref. ^15^) due to the increasing combustion of fossil carbon. This trend can cause the synchronized decrease of δ^13^C in plants^16^. Therefore, in addition to the shifting C4–C3 diet composition, the trend of δ^13^C in both C4 and C3 feeds will affect the temporal trends of livestock δ^13^C_CH4-ruminant_.

In this study, we establish a global, time-dependent dataset at national scale of the C3–C4 diet composition of domestic ruminants, the enteric methane emissions (F_CH4-ruminant_), and the flux weighted isotopic signature of the methane emissions (δ^13^C_CH4-ruminant_) over the period between 1961 and 2012. First, we separate the crop concentrate feeds consumed by ruminant vs. pigs and poultry using commodity and animal stocks statistics from FAOSTAT^9^. A simple feed model^17^ is used for this separation. Then we estimate the quantity of grass and occasional fodder and scavenged biomass based on ruminant energy requirement and grass-biomass use from previous studies, all with a distinction between C3 and C4. Then, using a relationship between δ^13^C_diet_ and δ^13^C_CH4-ruminant_ constructed in this study from δ^13^C_diet_ and δ^13^C_CH4-ruminant_ observations, we estimate the national- and time-dependent weighted isotopic signature of ruminant enteric methane emissions (δ^13^C_CH4-ruminant_) for the period of 1961–2012. Finally, we quantify the impact of the revised F_CH4-ruminant_ and δ^13^C_CH4-ruminant_ on δ^13^C_CH4-source_, and use a one-box model to quantify their effects on atmospheric CH_4_ concentration and δ^13^C_CH4-atm_. Table 1 provides a glossary of terms as used in this study.

**Table 1:** Glossary of terms as used in this study

Terms	Units	Explanations
δ^13^C_CH4_	‰	The ^13^C isotopic signature of methane; i.e., the ^13^C/^12^C-ratio of CH_4_ expressed in *δ*-notation relative to Vienna Pee Dee Belemnite (VPDB)-standard
δ^13^C_CH4-atm_	‰	The ^13^C isotopic signature of atmospheric methane
δ^13^C_CH4-sources_	‰	The ^13^C isotopic signature of methane from different source types, such as microbial sources, fossil-fuel sources, and biomass burning
δ^13^C_CH4-ruminant_	‰	The ^13^C isotopic signature of ruminant enteric fermentation methane emissions
δ^13^C_diet_	‰	The ^13^C isotopic signature of ruminant diet
DM	kg	Dry matter
*E*_CH4_	MJ (kg CH_4_)^−1^	The energy content of methane
*E*_GE_	MJ (kg dry matter)^−1^	The gross energy content of feeds
*F*_CH4-ruminant_	Tg CH_4_ yr^−1^	The annual ruminant enteric fermentation methane emissions
FCR	kg dry matter (kg live-weight gain or kg eggs production)^−1^	The feed conversion ratios
*f*_dressing_	%	The dressing percentage of livestock
*f*_intensity_	%	The farming intensity
*f*_DE_	%	The digestible fraction of gross energy contained in feeds (i.e., an indicator of digestibility)
GE	MJ	The gross energy intake/requirement by ruminants
ME	MJ	The metabolizable energy intake/requirement by ruminants
Q	kg dry matter	The total feed quantity used for different animal types
REM	%	The fraction of digestible energy available in diet used for maintenance
Weight	kg live-weight gain or kg eggs production	The total live weight of slaughtered animals or total weight of eggs production
*Y*_m_	%	The methane conversion factor

## Results

### Feeds for domestic livestock

**Fig. 1:**
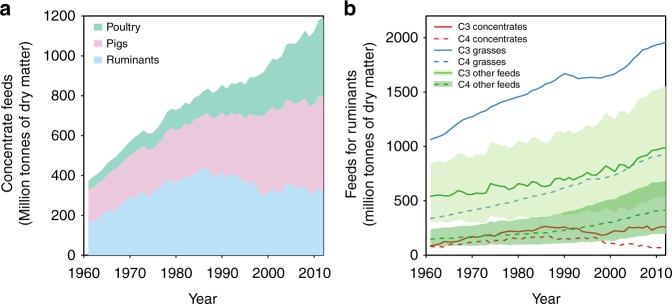
The changes in concentrate feeds for poultry, pigs, and ruminants, and in the composition of feeds for ruminants over the period of 1961–2012. The concentrate feeds for poultry, pigs, and ruminants are presented as stacked area chart in (**a**). The feeds for ruminants are concentrates (including all crop concentrate feed commodities for ruminants, C3-based or C4-based), grass (including C3 and C4 grasses), and other feeds (i.e., stover and occasional including C3 and C4 part) following ref. ^18^. The light and dark green lines in (**b**) show the mean amount of C3 and C4 other feeds consumed by ruminants estimated in this study derived from Monte Carlo ensembles (*n* = 1000) from the range of uncertainty reported on feed digestibilities (i.e., *f*_DE-s+o_, *f*_DE-concentrates_, and *f*_DE-grass_; see Methods section) and on the fraction of digestible energy available in diet used for maintenance (REMs; REM parameter values themselves dependent on *f*_DE_; see Methods). The green shaded areas show the 95% confidence interval of the estimates. Source data are provided as a Source Data file

Annual concentrate feed commodities for livestock increased from 373 million-tons dry matter (Mt DM) in 1961 to 1186 Mt DM in 2012 (Fig. 1a). The interannual variation of the feed could be due to many factors, such as market price of feed (supply-side) and livestock products (demand-side), climate (mainly supply-side), and even epidemic disease (demand-side). We will only focus on decadal average and long-term trend in this study. The feed model estimated that poultry and pigs consumed about half (53%) of the concentrate feeds in 1960s, the rest being for ruminants. In 2000s, 68% of the concentrate feeds were used for poultry and pig production against 32% for ruminants. The increasing share of concentrate feeds for poultry and pigs could be due to the larger increase in the production of poultry and pigs (increased by 11.9, 5.1, and 4.5-folds for poultry meat, eggs and pig meat production, respectively, between 1961 and 2012) compared with that of ruminant (increased by 2.1-fold between 1961 and 2012), and the farming intensity change. Annual concentrate feeds consumed by ruminants represented 213 Mt DM yr^−1^ in the 1960s, peaked at 402 Mt DM yr^−1^ in the 1980s and then decreased to 332 Mt DM yr^−1^ in the 2000s. The C4 concentrate feeds comprise 35% (1980s) to 37% (1990s) of the total concentrate feed commodities for livestock.

C3-based concentrate feeds constitute more than half of the ruminants’ concentrate feeds over the past five decades. The share of C4 in total ruminant’s concentrate feeds decreased from 43% in 1960s to 28% in 2000s. Concentrate feeds comprised only 8.0% [6.5–9.6%] of total dry matter consumption by ruminants in the 2000s, compared with 11.9% [9.9–13.9%] in the 1980s.

Grass-biomass is the largest share of ruminants’ dry matter consumption comprising about 63% [52–74%] of the total dry matter intake. It is noteworthy that the share of C4 grasses in grass feed increased significantly from 24.3% in 1960s to 31.3% in 2000s mainly due to the rapid C4 grass feed increase in Latin America and Caribbean (Supplementary Fig. 1).

Other feeds represent the second largest share of total dry matter consumption by ruminants, ranging from 25.8% [14.0–38.4%] in 1980s to 29.1% [15.3–42.3%] in 2000s (Fig. 1b). The share of C4 other feeds in total other feeds follows that of the grass feed, given the assumptions made in Methods (the C3:C4 ratio of other feeds the same as the ratio of grasses for each country).

In total, the C4 diet of ruminant weighted by the fraction of each type of feed increased from 25.2% [24.8–25.8%] in the 1960s to 30.3% [30.2–30.4%] in the 2000s.

### Relationship between δ^13^C_CH4-ruminant_ and δ^13^C_diet_

**Fig. 2:**
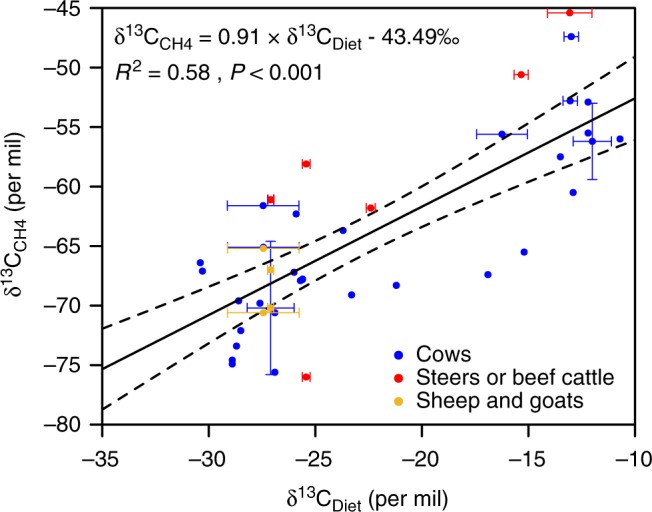
Linear regression between δ^13^C of diet (δ^13^C_diet_) and δ^13^C of CH_4_ from enteric fermentation of ruminants (δ^13^C_CH4-ruminant_). The dots denote 43 observations from six published documents^54–59^. δ^13^C_diet_ are either obtained directly from literature or calculated based on the feed composition and the δ^13^C uncertainties of different feed categories (Supplementary Table 1 and 2; see Methods for detail). In the latter case, to account for the effect of decreasing δ^13^C_CO2-atm_ on δ^13^C of feeds, we adjust the calculated δ^13^C_diet_ to the year when the δ^13^C_CH4-ruminant_ was measured assuming that the biomass used for feed grows in the same year as δ^13^C_CH4-ruminant_ measurement and follows the annual δ^13^C of atmospheric CO_2_. The solid line represents the best-fit correlation from the observations considering uncertainties of δ^13^C_diet_ and δ^13^C_CH4-ruminant_, and the dashed lines represent 95% confidence interval. The standard errors of the fitted slope and intercept are 0.12 and 2.86‰, respectively

Figure 2 shows the empirical relationship between δ^13^C_diet_ and δ^13^C_CH4-ruminant_ extracted from literature data (see Methods; Supplementary Tables 1 and 2). Here, we apply a linear regression, which results into the following equation:

1 $$\delta ^{13}C_{{\mathrm{CH4}}} = 0.91 \times \delta ^{{\it{13}}}C_{{\mathrm{diet}}} - 43.49 ‰ (R^2 = 0.58,p < 0.001)$$

with the standard errors of the fitted slope and intercept being 0.12 and 2.86‰, respectively (Fig. 2).

### Changes in *F*_CH4-ruminant_ and δ^13^C_CH4-ruminant_

**Fig. 3:**
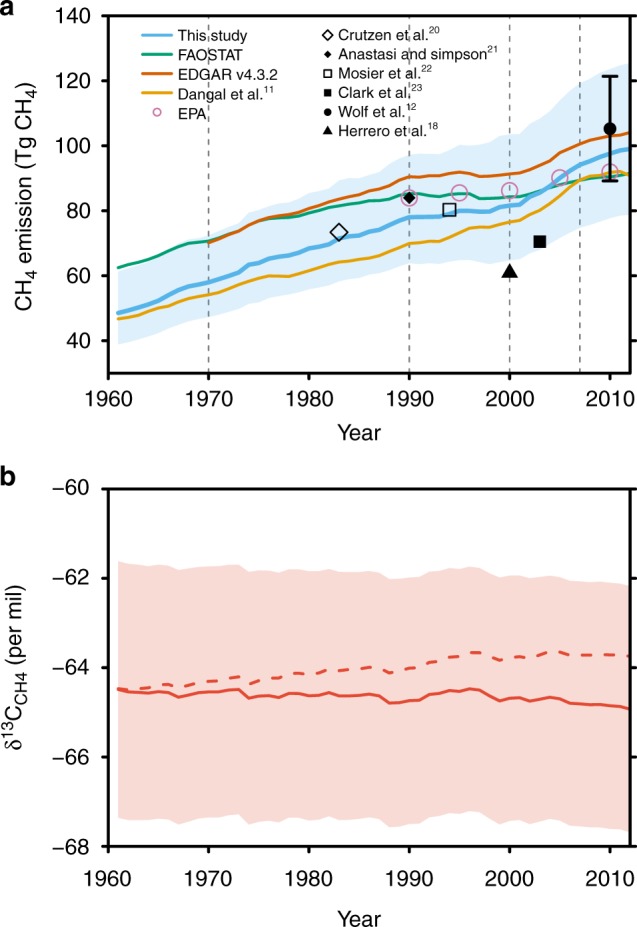
The changes in the global methane emissions from enteric fermentation of ruminants (*F*_CH4-ruminant_), and their globally weighted average isotopic signatures δ^13^C_CH4-ruminant_ over the period of 1961–2012. The blue (**a**) and red (**b**) shaded areas show the 95% confidence interval of the estimates of *F*_CH4-ruminant_ and δ^13^C_CH4-ruminant_, respectively. The red dashed line in (**b**) shows the δ^13^C_CH4-ruminant_ without accounting for the effect of δ^13^C_CO2-atm_ trend (see Methods). The dashed gray lines in (**a**) separate the periods that we consider the trend of *F*_CH4-ruminant_ in main text and Table 2. Source data are provided as a Source Data file

**Fig. 4:**
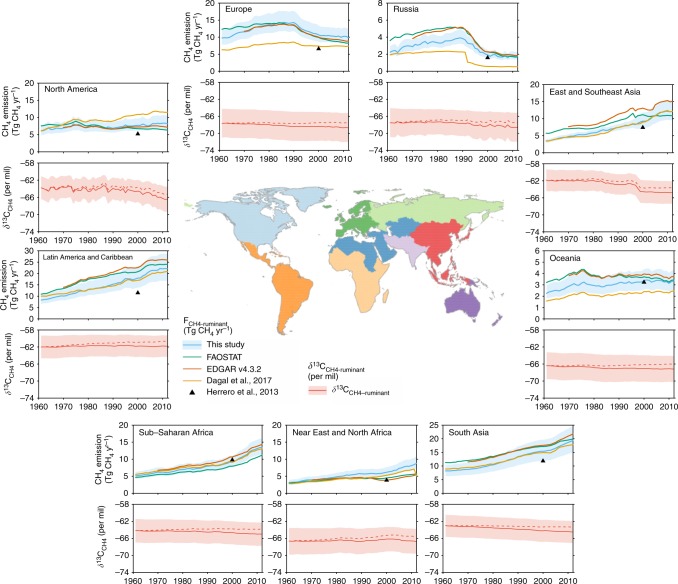
The changes in the regional methane emissions from enteric fermentation of ruminants (*F*_CH4-ruminant_), and their weighted average isotopic signatures δ^13^C_CH4-ruminant_ over the period of 1961–2012. The blue and red solid lines show the mean value of livestock emissions and their δ^13^C_CH4_ from this study, respectively. The blue and red shaded areas give the 95% confidence interval of those estimates. The red dashed lines show the δ^13^C_CH4-ruminant_ without accounting for the decreasing δ^13^C_CO2-atm_ trend (see Methods). Regions are classified following the definition of the FAO Global Livestock Environmental Assessment Model^66^. Western and eastern Europe are combined as Europe. The vertical-scale of the regional methane emissions has been adjusted so that the changes can be easily seen

We estimated that global *F*_CH4-ruminant_ has doubled from 48.5 ± 5.6 Tg CH_4_ yr^−1^ (mean ± 1-sigma standard deviation) in 1961 to 99.0 ± 11.7 Tg CH_4_ yr^−1^ in 2012 (Fig. 3a). The emissions’ increase mainly took place in the Latin American and Caribbean countries (+13.8 ± 2.0 Tg CH_4_ yr^−1^), East and Southeast Asia (+8.8 ± 1.3 Tg CH_4_ yr^−1^), Sub-Saharan Africa (+8.4 ± 0.7 Tg CH_4_ yr^−1^), Near East and North Africa (+5.6 ± 0.6 Tg CH_4_ yr^−1^), and South Asia (+11.1 ± 1.4 Tg CH_4_ yr^−1^) (Fig. 4). By contrast, *F*_CH4-ruminant_ decreased in Europe and Russia during the period of 1990–2012 by 31% and 54%, respectively, making the emissions of 2012 lower than those of 1961 in these two regions.

The global mean δ^13^C_diet_ decreased a little from −23.05‰ [−25.45 to −20.66‰] in 1961 to −23.53‰ [−25.74 to −21.31‰] in 2012 (data not shown). This global diet change together with the decreasing δ^13^C of feeds due to decreasing δ^13^C_CO2-atm_ caused marginal change in the global mean δ^13^C_CH4-ruminant_ (ranging from −64.49‰ [−67.36 to −61.62‰] in 1961 to −64.93‰ [−67.68 to −62.17‰] in 2012; Fig. 3b). However, δ^13^C_CH4-ruminant_ has noticeable changes in several regions. There are δ^13^C_CH4-ruminant_ increases in Near East and North Africa, Latin America, and Caribbean. Decreases in δ^13^C_CH4-ruminant_ are found in North America since 1990, and in East and Southeast Asia during 1992–1996 caused by an increased reliance of C3 vs. C4 concentrates feed (in relative share; Supplementary Fig. 1).

**Fig. 5:**
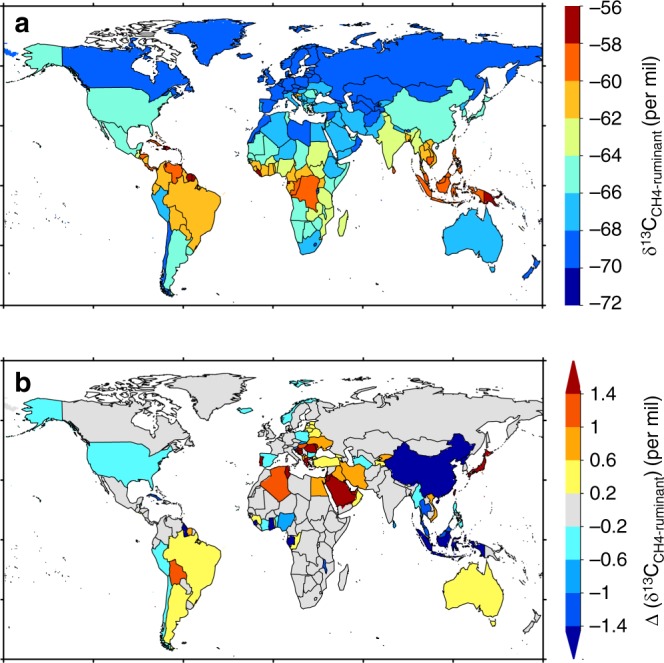
Country average δ^13^C_CH4-ruminant_ in 2000s, and the changes in δ^13^C_CH4-ruminant_ between 1960s and 2000s (∆ (δ^13^C_CH4-ruminant_)). The ∆ (δ^13^C_CH4-ruminant_) is here calculated from the diet shift only (i.e., changes in the relative C3–C4 fraction in feeds) without accounting for decreasing δ^13^C_CO2-atm_ incorporated in the biomass of grazed plants (see Methods). A positive ∆ (δ^13^C_CH4-ruminant_) indicates an increase of δ^13^C_CH4-ruminant_ from 1960s to 2000s from a higher fraction of C4 in the diet

Figure 5a shows the global distribution of national average δ^13^C_CH4-ruminant_ in 2000s. Countries in tropical regions tend to have isotopically heavier ruminant methane emissions (less negative δ^13^C_CH4-ruminant_) due to a higher C4 plants proportion in the diet of animals. Country-level δ^13^C_CH4-ruminant_ due to diet shift only shows large changes in opposite sign (heavier or lighter; Fig. 5b). Within the major livestock producing countries, δ^13^C_CH4-ruminant_ decreased by −0.3‰ and −1.9‰ in the United States and China, respectively. In these two countries, the increase in poultry and pig numbers consumed most crop feeds, including maize, so that only few C4 crops feed became allocated to ruminants. The lower fraction of C4 diet explained the decrease of δ^13^C_CH4-ruminant_ in these two countries. In Indonesia and Malaysia, the average δ^13^C_CH4-ruminant_ showed a strong decrease of −1.8‰ and −1.2‰, respectively. More C4 crop feed was used there to feed the increasing poultry numbers (i.e., less C4 crops left for ruminant). By contrast, δ^13^C_CH4-ruminant_ significantly increased from the 1960s to the 2000s in Brazil (+0.3‰), Argentina (+0.5‰) and Australia (+0.5‰), which are major livestock producing countries. This increase in the source signature is explained by the combined effect of increased C4 crop feed and C4 pasture grazing.

## Discussion

Global livestock feed data, including concentrates, grasses, stover, and occasional feeds, are available only for the year 2000^18^. In this study, we reconstruct the consumption of concentrate crop feeds consumed by ruminant, the grazing of grass-biomass, and inferred the consumption of stover and occasional (other feeds) biomass as a residual to meet the metabolic energy requirement of the whole ruminant production sector over the period of 1961–2012 (Fig. 1). For 2000, we estimate that ruminants consumed 33% of the total concentrate feeds (317 Mt), 11% higher than the value given by ref. ^18^ (284 Mt). The difference could be due to the uncertainty in the feeds estimate for poultry and pigs in this study. The uncertainty could come from the feed conversion ratios (FCR) used here. For developed countries, we applied the FCR of poultry production derived from the industrial broiler system of the United States (intensive system with relatively low FCR). The FCRs of developing countries are assumed to be 20% higher than those of developed countries (derived from the FCR difference for pig production between the United States and China). On one hand, the FCR of poultry production other than broiler (like other types of chicken, duck, and turkey) is assumed to be the same as that of industrial broiler, which could bring uncertainty to the feed estimates. On the other hand, industrial and smallholder systems are assumed to have the same FCR for production in the simple feed model, due to the lack of the FCR information for smallholder system. Usually, smallholder system tends to have higher FCR than industrial system. This will cause the low feed requirement estimated by the simple feed model. In addition, the uncertainty could be due to the assumed proportion of backyard production that did not receive feed commodities collected in statistics (see Methods). In a simulation assuming that all poultry and pig productions receive feed commodities, the simple feed model estimates poultry and pigs consumed 78% of the total concentrate feeds (1013 Mt), with 22% eaten by ruminants (110 Mt).

Nevertheless, concentrate feeds comprised <10% of the total dry matter consumption by ruminants in the 2000s. The grass feed fraction in 2000 used in this study was directly derived from ref. ^18^. Stover and occasional feed used by ruminants is clearly the most uncertain term of the feed equation. They were estimated to be 1098 Mt in average for 2000, close to the estimate of ref. ^18^ (1131 Mt). But our estimates have large uncertainty ranging from 477 to 1976 Mt (95% confidence interval) due to the large uncertainty in digestibility of feeds. In summary, the simple feed model and assumptions on feed digestibility of ruminants’ feed can generally reproduce livestock feed consumptions at global scale well compared with ref. ^18^.

In this study, annual *F*_CH4-ruminant_ was calculated based on IPCC Tier 2 methods. Compared with Tier 1 method that used the livestock population data and default emission factors, Tier 2 approach uses a more detailed country-specific data on gross energy intake and methane conversion factors for specific livestock categories, allowing the consideration of diet quantity and quality^13^. Table 2 shows the comparison of the global *F*_CH4-ruminant_ estimated in this study and those from previous studies for the contemporary period (1980–2012; see also Fig. 3a). Our global estimates are generally within the range of previous estimates using IPCC Tier 1 and Tier 2 methods^9–12,19–23^. However, discrepancies exist between estimates using different methods and input data. IPCC Tier 2 method considers gross and net energy requirement and associated feed intake by different livestock categories (cattle, buffaloes, sheep, and goats) and sub-categories (dairy of non-dairy), and digestible energy availability of the diet (i.e., feed digestibility). For IPCC Tier 1 method, default emission factors for non-cattle livestock are from literature with indicated live-weight (see Tables 10.10 of ref. ^13^, Vol. 4, Chapter 10, pp. 10.28), and default region-specific emission factors for cattle used in a Tier 1 method (Tables 10.11 of ref. ^13^, Vol. 4, Chapter 10, pp. 10.29) are derived in fact from Tier 2 method and the data in Tables 10 A.1 and 10A.2 of ref. ^13^, Vol. 4, Chapter 10, pp. 10.72–10.75, which embodies livestock characteristics adjudged by expert opinions. Expert opinion necessarily limits confidence in those default emission factors to be representative of all cattle in large regions such as Africa, Middle East, and Asia. Compared with Tier 1 method, Tier 2 method (if related active data like body weight and feed digestibility are used) should allow a more accurate estimate of feed intake which is an important variable in estimating methane production from enteric fermentation (ref. ^13^, Vol. 4, Chapter 10, pp. 10.10). Reference ^18^, using Tier 3 approach with herd dynamics (i.e., age of first calving, replacement, and live-weight gain) and detailed parameterization of feed intake and rumen fermentation, estimated lower emissions in the year 2000 than all other studies (including our study; Fig. 3a). The highest emission comes from ref. ^12^ based on Tier 1 and accounting for recent changes in animal body mass, feed quality and quantity, milk productivity, and management of animals and manure.

**Table 2:** Comparison of the global methane emissions from enteric fermentation of ruminants (*F*_CH4-ruminant_) and its trend

Year	This study (Tg CH_4_ yr^−1^)	Estimates from previous studies (Tg CH_4_ yr^−1^)	Method/emission factors	Sources
Global *F*_CH4-ruminant_				
1983	71.5 ± 7.8	73.4	IPCC Tier 2	Crutzen et al.^20^
1990	78.0 ± 8.4	84	IPCC Tier 1	Anastasi and Simpson^21^
1994	79.0 ± 8.6	80.3	IPCC Tier 1	Mosier et al.^22^
2000	81.6 ± 9.6	60.9	IPCC Tier 3	Herrero et al.^18^
		86.3	Mixed IPCC Tiers^a^	EPA, 2012^19^
		84.3	IPCC Tier 1	FAOSTAT^9^
		91.3	Hybrid IPCC Tier 1	EDGAR v4.3.2^10^
		76.5	IPCC Tier 2	Dangal et al.^11^
2003	85.6 ± 10.0	70.5	IPCC Tier 2	Clark et al.^23^
2000s	88.9 ± 10.4	96.0 ± 14.7	Revised IPCC Tier 1	Wolf et al.^12^
		83.8	IPCC Tier 2	Dangal et al.^11^
2010	97.6 ± 11.4	92.0	Mixed IPCC Tiers^a^	EPA, 2012^19^
		90.4	IPCC Tier 1	FAOSTAT^9^
		103.1	Hybrid IPCC Tier 1	EDGAR v4.3.2^10^
		105.3 ± 16.1	Revised IPCC Tier 1	Wolf et al.^12^
		91.8	IPCC Tier 2	Dangal et al.^11^
Trend of global *F*_CH4-ruminant_				
Period	This study (Tg CH_4_ yr^−2^)	Estimates from previous studies (Tg CH_4_ yr^−2^)	Method/emission factors	Sources
1961–2012	0.92 ± 0.12	0.50	IPCC Tier 1	FAOSTAT^9^
		0.86	IPCC Tier 2	Dangal et al.^11^
1970–1989	0.95 ± 0.09	0.69	IPCC Tier 1	FAOSTAT^9^
		0.94	Hybrid IPCC Tier 1	EDGAR v4.3.2^10^
		0.70	IPCC Tier 2	Dangal et al.^11^
1990–1999	0.33 ± 0.14	−0.14	IPCC Tier 1	FAOSTAT^9^
		0.05	Hybrid IPCC Tier 1	EDGAR v4.3.2^10^
		0.74	IPCC Tier 2	Dangal et al.^11^
2000–2012	1.61 ± 0.20	0.64	IPCC Tier 1	FAOSTAT^9^
		1.19	Hybrid IPCC Tier 1	EDGAR v4.3.2^10^
		1.22	Revised IPCC Tier 1	Wolf et al.^12^
		1.47	IPCC Tier 2	Dangal et al.^11^

Values from this study are shown as mean ± 1-sigma standard deviation

^a^U.S. EPA dataset is based on IPCC Tier 1 calculations supplemented with country-reported inventory data (ref. ^19^, pp.1), with most of the enteric CH4 emissions being from country-reported inventory data (Appendices of ref. ^19^, pp. G-8 to G-9). Given the fact that a majority of the reported data were derived from the UNFCCC flexible query system using higher IPCC Tiers, we called the method used by U.S. EPA data Mixed IPCC Tiers

The linear trend of global *F*_CH4-ruminant_ found in this study (0.89 ± 0.11 Tg CH_4_ yr^−2^) is much higher than that from FAOSTAT^9^ (0.50 Tg CH_4_ yr^−2^), and is not different from that of ref. ^11^ (0.86 Tg CH_4_ yr^−2^) for the period of 1961–2012. For the period of 1970–1989, we estimate a trend of *F*_CH4-ruminant_ (0.95 ± 0.09 Tg CH_4_ yr^−2^) similar to that from EDGAR v4.3.2^10^ (0.94 Tg CH_4_ yr^−2^) and high than those from FAOSTAT^9^ and ref. ^11^ (Table 2). During 1990s, the emission trend from this study (0.33 ± 0.14 Tg CH_4_ yr^−2^) is larger than those from FAOSTAT^9^ (−0.14 Tg CH_4_ yr^−2^) and EDGAR v4.3.2^10^ (0.05 Tg CH_4_ yr^−2^), but lower than that in ref. ^11^ (0.74 Tg CH_4_ yr^−2^). For the period of 2000–2012, we estimate a trend of (1.61 ± 0.20 Tg CH_4_ yr^−2^) which is higher than those from FAOSTAT^9^, EDGAR v4.3.2^10^ and ref. ^12^, but similar to that from ref. ^11^ (1.47 Tg CH_4_ yr^−2^) using IPCC Tier 2 method. The differences of the trend estimates could come from several factors such as trends in milk yield and carcass weight, the statistics of ruminant numbers, and the assumptions of digestibility.

First, the trends from FAOSTAT^9^ are lower than all other estimates, mainly due to the fact that the estimate of FAOSTAT did not consider any trend of carcass weight and milk productivity. The increasing trend of carcass weight and milk productivity resulted into higher emissions per unit livestock as in all estimates other than those from FAOSTAT^9^ (see Methods). The EDGAR v4.3.2 inventory^10^ applies IPCC Tier 1 methods, but uses country-specific milk yield and carcass weight trend for cattle emissions (not for other animal types like sheep and goats; i.e., a hybrid Tier 1 method). This study and ref. ^11^ both account for country-specific milk yield and carcass weight trend for all ruminants.

Second, ruminant livestock numbers used in this study and EDGAR v4.3.2^10^ are all from FAOSTAT^9^, while ref. ^11^ used additional data from subnational administrative regions in several countries, including the United States, Australia, Brazil, Canada, China, and Mongolia. This will affect the *F*_CH4-ruminant_ and its trend.

Third, the method used to calculate *F*_CH4-ruminant_ can affect the trend estimate. Time invariant default emission factors of IPCC Tier 1 method were used by FAOSTAT^9^ and EDGAR v4.3.2^10^. This study and ref. ^11^ applied IPCC Tier 2 method considering gross and net energy requirement, and digestible energy availability of the diet (i.e., feed digestibility). But there are differences in the digestibility used. Here, we consider different digestibility according to time varying changes in the different feed types in each country (Eq. (10)) while ref. ^11^ used regional average feed digestibility derived from ref. ^24^ across the history.

Using IPCC Tier 2 method, our estimate and that from ref. ^11^ both give a large increase in emissions between 2002–2006 and 2008–2012, of 8.4 Tg CH_4_ yr^−1^ in ref. ^12^ (including manure management emissions; an increase of 6.9 Tg CH_4_ yr^−1^ for enteric emissions only), 8.6 Tg CH_4_ yr^−1^ in ref. ^11^ and 9.4 ± 2.0 Tg CH_4_ yr^−1^ in this study. These increases are larger than the estimate of in previous studies^9,10,19^ (an increase ranging from 3.6 to 6.5 Tg CH_4_ yr^−1^; Table S5 of ref. ^8^ for details). This suggests a larger contribution of enteric emissions to the recent increase in global methane emissions, and also a larger contribution to the recent decrease in the *δ*^13^C of atmospheric methane (lighter δ^13^C_CH4-atm_) given the lighter δ^13^C_CH4-ruminant_ than δ^13^C_CH4-atm_ (see Methods). It should be noted that there is larger trend of *F*_CH4-ruminant_ in the period of 1999–2006 (1.7 ± 0.2 Tg CH_4_ yr^−2^) and that in the period of 2008–2012 (1.0 ± 0.1 Tg CH_4_ yr^−2^). The plateau of atmospheric methane concentration observed between late-1990s and mid-2000s therefore comes from other sources with larger emission decrease^3^ or/and from sink variability^5^.

At regional scale, *F*_CH4-ruminant_ estimated in this study is generally in agreement with those from FAOSTAT^9^ (using IPCC Tier 1 method), EDGAR v4.3.2^10^ (using a hybrid IPCC Tier 1 method with partial consideration of the trends in livestock productivity), and ref. ^11^ (using IPCC Tier 2 method; Fig. 4). One major difference comes from Latin America and Caribbean, where we estimate a lower *F*_CH4-ruminant_ than those from FAOSTAT^9^ and EDGAR v4.3.2^10^. Our estimate is close to that from ref. ^11^ using IPCC Tier 2 method, and higher than that from ref. ^18^ using IPCC Tier 3 method. Differences can also be found among our estimates and those from ref. ^11^ and ref. ^18^ in North America, Europe, Russia, and Oceania. This could be due to the methods (IPCC Tier 1, 2 or 3 method), statistics of livestock numbers and live weight, and regional digestibility. For example, low estimates from ref. ^11^ in Europe, Russia, and Oceania could be due to the high feed digestibility used (derived from Table B13 of ref. ^24^). In Russia, the difference is due to the low ratio of non-dairy to dairy cattle in ref. ^11^ (data not shown), as non-dairy cattle emission intensity is higher than that of dairy cattle. Large differences can be found for emissions of the United States using various methods and statistics. For example, ref. ^25^ conducted an inventory of emissions in 1990s using subnational cattle numbers of different sub-groups, measured methane emission from each sub-group, and predominant type of diets. They reported that U.S. cattle emitted 6.6 Tg CH_4_ in 1998, which is similar to the estimates of this study (6.7 ± 0.8 Tg CH_4_ for all ruminants). Reference ^12^ recently reported U.S. enteric emission of 6.6 ± 1.0 Tg CH_4_ in 2012 using revised emission factors, which is similar to that of ref. ^26^ (6.2 Tg CH_4_) and our estimate (7.4 ± 0.9 Tg CH_4_). Using subnational livestock data and IPCC Tier 2 method, ref. ^11^ reported emissions larger than other estimates (9.4 Tg CH_4_ in 1998, and 9.9 Tg CH_4_ in 2012; also in Fig. 4).

Tropical and sub-tropical regions experienced much higher population growth than temperate regions in the past five decades^27^. People’s diet in tropical and sub-tropical regions was also shifted toward a more animal-based protein consumption^9^. Population growth and diet shift together resulted in a larger increase in ruminant numbers and feed requirements in regions like Latin America and Caribbean, Sub-Saharan Africa, Near East and North Africa, South Asia, and East and Southeast Asia (Supplementary Fig. 1). In addition to the local consumption, international trade also contributes to the feed requirement and *F*_CH4-ruminant_ increase in regions like Latin America and Caribbean. Net export of ruminant meat increase from 0.65 million ton in 1961 to 2.1 million ton in 2012^9^. Given the fact that concentrate feeds comprised only around 10% of the total ruminant feeds (see Results section), the C4 diet fraction in ruminant diet mainly depends on C3–C4 distribution of local feeds. As a result, larger increase in ruminants over tropical and sub-tropical regions (where C4 plant is relatively more dominant) compared with that of temperate regions, is the main cause of the global increase in the C4 diet fraction in the ruminant diet between 1960s and 2000s.

Latin America and Caribbean has the highest C4 diet fraction (Supplementary Fig. 1), and also has the largest increase in C4 diet fraction (from 43.9% [43.5–45.1%] in 1961 to 54.6% [54.2–54.9%] in 2012. This region makes the major contribution to the global increase in C4 diet fraction. The C4 diet also comprises a significant part of ruminant diet in the United States, Sub-Saharan Africa, South Asia, and East and Southeast Asia. Local C4 feeds are the dominant component of the C4 diet in these regions except the United States and China. C4 concentrates comprise 19% and 21% of the total ruminant feeds in the United States and China during the 1980s, respectively. However, due to the fast growth of poultry and pig numbers, more C4 concentrates in these two countries are used for poultry and pigs with less for ruminants simulated by our simple feed model. Thus, the C4 diet fractions in these two countries decreased in the past two decades.

The regional evolution of *F*_CH4-ruminant_ follows the regional growth of ruminant numbers and feed consumptions, which is also a result of population growth and diet shift. In opposite to the vast *F*_CH4-ruminant_ increase in most regions, large decreases in *F*_CH4-ruminant_ are found in Europe and Russia between 1990 and 2012. For eastern Europe and Russia, the collapse of the Former USSR in early 1990s caused large decrease in livestock numbers and thus *F*_CH4-ruminant_. In western Europe, the decrease in livestock numbers and *F*_CH4-ruminant_ comes from a series of policies: (1) the European Union has provided various incentives to farmers through the Common Agricultural Policy (CAP^28^) since 1962 to avoid the negative side-effects of some farming practices, and has shifted the incentives from price support to direct aid payments to farmers who withdraw land from production in 1992 (thus reduced livestock stocking levels); (2) in 1984, the European Community introduced milk production quotas that contributed to a reduction in the dairy cow population; (3) in 1991, the Nitrates Directive (91/677/EEC^29^) restricted the application of animal manure in nitrate vulnerable zones to a maximum of 170 kg N ha^−1^, which caps livestock density in pasture at some 1.7 livestock unit per ha (Annex 1 in ref. ^30^).

Large scatters exist in the data shown in Fig. 2. Due to lack of a more plausible relationship, the choice of linear relationship is a reasonable assumption and an interpretation of the data. Interestingly, the resulted equation is similar to the usual isotope effects modeled with first-order kinetics^31^, which is given by:

2 $$\alpha = \frac{{R_{{\mathrm{diet}}}}}{{R_{{\mathrm{CH4}}}}}$$

where *α* denotes the isotope effects, where *α* ≅ 1 and is denoted as (1+*ε*); *R*_diet_ and *R*_CH4_ denote the 13C/12C molar ratio of diet (reactant) and ruminant enteric CH_4_ emissions (products of reaction), respectively. Using δ^13^C_diet_ and δ^13^C_CH4_ (*R*_diet_/*R*_VPDB-standard_−1 and *R*_CH4_/*R*_VPDB-standard_−1, respectively) instead of *R*_diet_ and *R*_CH4_, the Eq. (2) can be transformed to:

3 $$1 + \varepsilon = \frac{{1 + {\mathrm{\delta }}^{13}{\mathrm{C}}_{{\mathrm{diet}}}}}{{1 + {\mathrm{\delta }}^{13}{\mathrm{C}}_{{\mathrm{CH4}}}}}$$

Eq. (3) can lead to:

4 $${\mathrm{\delta }}^{13}{\mathrm{C}}_{{\mathrm{CH4}}} = \frac{{{\mathrm{\delta }}^{13}{\mathrm{C}}_{{\mathrm{diet}}}}}{{1 + \varepsilon }} + \frac{\varepsilon }{{1 + \varepsilon }}$$

which is usually linearized to:

5 $${\mathrm{\delta }}^{13}{\mathrm{C}}_{{\mathrm{CH4}}} = {\mathrm{\delta }}^{13}{\mathrm{C}}_{{\mathrm{diet}}} + \varepsilon$$

Given the uncertainties in our regression coefficient for slope (0.91 ± 0.12), our linear regression (Eq. (1)) is compatible with Eq. (5) with a *ε* = 43.49 ± 2.86‰, where the resulted *ε* can be seen as the isotopic discrimination factor of the fermentation processes of livestock rumen. The resulted equation reflects the biochemical reactions for stable isotopes, which always produce depleted products (^13^C) while enriching the remaining substrates (^12^C) owing to the preference of enzyme systems to use lighter isotope substrates ^12^C.

Different δ^13^C_CH4-ruminant_despite the same δ^13^C_diet_ was found in the collected data (i.e., the large vertical scatter in Fig. 2). This isotopic variability could be due to the differences in first, exact feed composition (e.g., C3 feed: barley, wheat, soybean, alfalfa, straw, or C3 grass; C4 feed: maize grain, maize silage, or C4 grass), second, variation of feed δ^13^C in space and time, plant isotopic fractionation related to water use efficiency (WUE), and growing season δ^13^C_CO2-atm_ when CO_2_ is fixed by plants and incorporated into biomass, third, energy content of feed, and fourth, the different ruminant species (cow, steer, goat, or sheep). For example, the feed composition given by the literature is sometimes coarse for some data points (i.e., points reporting a general C3 or C4 diet in Supplementary Table 2). In this study, we estimate δ^13^C_diet_ using δ^13^C data collected for different feed categories, considering their uncertainty, and adjust them to the sampling year of δ^13^C_CH4-ruminant_ using an adjustment factor derived from historical changes in δ^13^C_CO2-atm_ from ref. ^15^ (see Methods). This adjustment partly accounts for the δ^13^C_diet_ variability caused by different feed composition and different years of measurements. Spatial and temporal variability of δ^13^C in feed plants cannot be addressed given the sparse data. Energy content of feed might also affect the isotopic variability, through its relation to gut microbes conversion of intake into CH_4_. Given the fact that gut microbes preferably break down ^12^C components, high energy content of feed could potentially increase the conversion to CH_4_ by gut microbes, and cause heavier enteric CH_4_ emissions. Ruminant species and even the characteristics of individual animal could also be a source of the variations in δ^13^C_CH4-ruminant_. For example, as shown in Fig. 2, between animals fed by diet with similar δ^13^C_diet_, the δ^13^C_CH4-ruminant_ can vary between different ruminant species (shown with different colors in Fig. 2) and within the same species (the dispersion of δ^13^C_CH4-ruminant_ with similar δ^13^C_diet_ shown with the same color in Fig. 2). The variation in δ^13^C_CH4-ruminant_ caused by different ruminant species was also observed in a study using identical feed conditions for cows, sheep, and camels^32^, while the authors did not detect significant δ^13^C_CH4-ruminant_ variation within each species.

We estimate that the annual mean δ^13^C_CH4-ruminant_ slightly decreased from −64.49‰ [−67.36 to −61.62‰] in 1961 to −64.93‰ [−67.68 to −62.17‰] in 2012. This net small trend over 50 years is the result of two opposite mechanisms the increasing proportion of C4 grass and feeds (occasional and stover) consumed by ruminants (Fig. 1), and the decreasing δ^13^C_CO2-atm_ that is incorporated in the biomass consumed by ruminants. The first mechanism tends to increase δ^13^C_diet_ (less negative) of +0.74‰ over the last 50 years, while the second effect causes a decrease of δ^13^C_diet_ of 1.18‰. Note that the magnitude of the decreasing trend of δ^13^C of biomass is not only parallel with the decreasing δ^13^C_CO2-atm_, but it is also controlled by trends of WUE over that period. There is evidence for an increase of WUE in temperate, boreal, and tropical forests^33–36^ over the last 50 years partly attributed to increasing CO_2_ in the atmosphere, but less so for C3 crops and grasses consumed by livestock^37^. Thus, in absence of direct observations at appropriate spatial and temporal scales to confirm an increased WUE for C3 plants consumed by livestock and given the fact that C4 plants are not expected to increase their WUE under elevated CO_2_, we assumed conservatively that WUE is constant, which may overestimate the negative trend of δ^13^C_CH4-ruminant_ reflecting the trend of δ^13^C_CO2-atm_.

Our estimate is significantly lower than the data compilation from ref. ^5^ (−61‰), higher but within the uncertainty range of ref. ^7^ (−65.4 ± 6.7‰ with a median of −67.1‰), and at the upper bound of the 1-sigma standard deviation of value estimated by ref. ^6^ (−66.8 ± 2.8‰). The values given in ref. ^7^ from δ^13^C_CH4-ruminant_ obtained in local studies were not weighted by the proportion of C3- versus C4-eating ruminants. The value estimated by ref. ^6^ is derived from data-driven δ^13^C_CH4-ruminant_ estimates of −54.6 ± 3.1‰ for C4 plant-based diet, and of −69.4 ± 3.1‰ for C3 plant-based diet, and a global weighted average C4 diet fraction ranging from 1.5 to 19.6% (uniform distribution). The range is simply estimated by using the estimated C4 emission fraction of the United States (19.6%) as a global upper bound (i.e., the rest of the world have the same C4 emission fraction as the United States) and zero C4 emission fraction except the United States as a global lower bound (i.e., 1.5%). As comparison, our estimate is based on first, the refined national C3:C4 feed fraction and its associated δ^13^C_diet_ considering the observation-based uncertainties in the δ^13^C of different feed categories and the impact of decreasing δ^13^C_CO2-atm_ on the δ^13^C of feeds, and second, a data-driven relationship between δ^13^C_diet_ and δ^13^C_CH4-ruminant_ (see Methods). For the United States, we estimated a C4 feed fraction of 22.2 ± 0.3% for the period of 1980–2012, which is higher but within the uncertainty of the fraction assumed by ref. ^6^ (19.6 ± 5.9% during 1980–2012).

In this study, we assess the uncertainties of the composition and the δ^13^C of ruminant diet (δ^13^C_diet_), ruminant enteric methane emission (*F*_CH4-ruminant_), and its weighted δ^13^C (δ^13^C_CH4-ruminant_) through Monte Carlo ensembles (*n* = 1000) considering the uncertainties of the parameters used in calculation (see Methods and Supplementary Table 3). The parameters’ uncertainties considered here include the feed digestibility, the fraction of digestible energy available in diet used for maintenance (REMs), the methane conversion factor (*Y*_m_), the δ^13^C of feed categories, and the fitted linear regression between δ^13^C_diet_ and δ^13^C_CH4-ruminant_ constructed from observations.

We also acknowledge other uncertainties that are beyond our capacity of more precise estimation at current stage.

When estimating concentrate feeds intake by pigs and poultry, we account for the national livestock productivity, the different feed conversion ratio (converting production to feed requirement) and farming intensity (industrialized vs. backyard production) for developing and developed countries. Uncertainties in the feed conversion ratio have been discussed above. Besides, there could be uncertainties from other aspects. For example, the simple feed model is based on diet composition of Germany, which could cause inevitable uncertainties in the C3–C4 feed composition of pigs and poultry, and further affects uncertainties in C3–C4 concentrate feed for ruminants. There are also uncertainties in our assumptions on the logistic intensification and constant farming intensity of pig and poultry production in developing and developed countries, respectively, which will affect the time evolution of the concentrate feeds consumption by pigs and poultry. However, these uncertainties are currently not accessible due to lack of national-specific information on diet and farming intensity, and their historical changes.

In this study, we account for the impact of the global annual mean δ^13^C_CO2-atm_ trend on the trend of δ^13^C of feed, δ^13^C_diet_ and δ^13^C_CH4-ruminant_, while the potential effects of the latitudinal gradient and seasonality of δ^13^C_CO2-atm_ (http://scrippsco2.ucsd.edu/graphics_gallery/isotopic_data/global_stations_isotopic_c13_trends) on the δ^13^C of feed (plants) are not considered. C4 photosynthesis is competitive under low atmospheric CO_2_ or high temperature/low water availability^14^. We constructed gridded C3–C4 grass (and other local feeds) distributions based on growing season temperature^38^. The impact of the rising CO_2_ (~76 ppm during 1961–2012) on the C3–C4 grass distribution is not considered due to lack of evaluated global estimation. Based on leaf photosynthetic rates, one may expect an increase in C3 grass species due to elevated CO_2_, thus a lower δ^13^C_diet_ and a lower δ^13^C_CH4-ruminant_. But long-term CO_2_ enrichment experiments have not consistently shown a decrease of C4 species in mixed grasslands^39^ suggesting ecosystem-level mechanisms that maintain a fitness of C4 plants to elevated CO_2_. In addition, δ^13^C of C3 plants may have a dependence on mean annual precipitation^40,41^, which could potentially affect the spatial patter of the feed δ^13^C. However, a through meta-analysis on the effect on crops and grasses is needed before such relationship can be applied for assessing δ^13^C of feeds.

In the following section, we examine more in details the impact of our revised estimates *F*_CH4-ruminant_ and δ^13^C_CH4-ruminant_ on the trends of global atmospheric CH_4_ concentration and its isotopic composition using the time-dependent one-box model of the CH_4_ budget from refs. ^42,43^ (see Methods). A baseline simulation was run using the bottom-up reconstructions of the methane sources and tuning the historical atmospheric sink history for ^12^CH_4_ and ^13^CH_4_ to match atmospheric observations. Enteric methane emissions from EDGAR v4.3.2 were used in this baseline simulation as it is the most widely used prior inventory of atmospheric inversions^2^. Three perturbed runs were conducted to separate the effects of revised *F*_CH4-ruminant_, revised δ^13^C_CH4-ruminant_ and revised δ^13^C_CH4-ruminant_ changes between 1961 and 2012, respectively (see Methods for details in the model and the simulations). The purpose of the box-model simulations is to show the impact of our new estimates of livestock emissions on atmospheric trends, not to provide closure or re-analysis of recent changes in the budget for which adjustment of other sources (wetlands, fossil fuels) would be required.

**Fig. 6:**
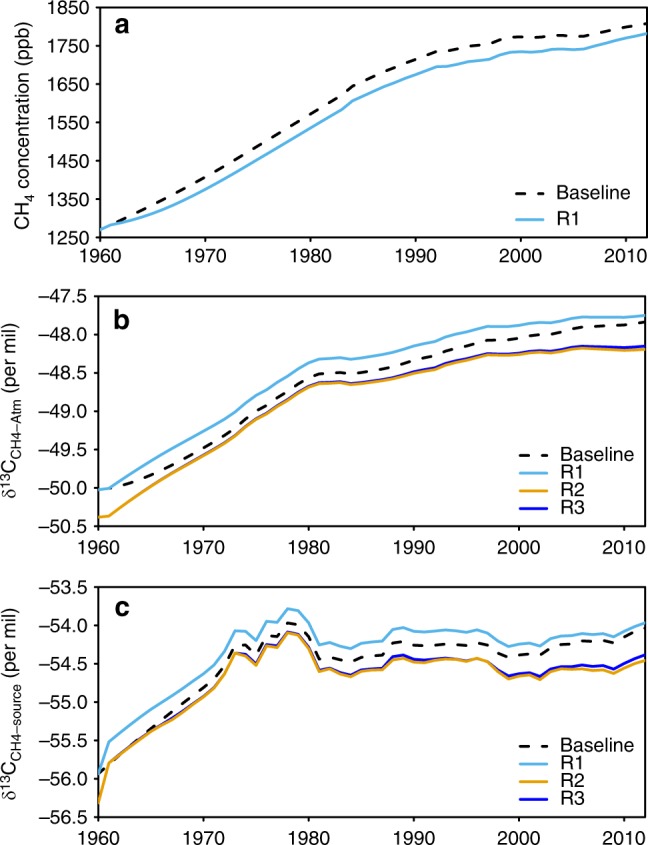
Global box model simulations of atmospheric CH_4_ concentration, the box model results on δ^13^C_CH4-atm_, and the isotopic source signature weighted by all sources (δ^13^C_CH4-source_). In the simulation of R1, only the revised *F*_CH4-ruminant_ is used and δ^13^C_CH4-ruminant_ set to default value of −62‰ previously used by ref. ^5, 43^; In R2, the revised *F*_CH4-ruminant_ and δ^13^C_CH4-ruminant_ are used (see Methods). R3 is the same as R2 but with constant δ^13^C_CH4-ruminant_ at −64.49‰ for the period of 1961–2012. The differences between R1 and baseline are the effects of the revised *F*_CH4-ruminant_; the differences between R2 and R1 are the effects of the revised δ^13^C_CH4-ruminant_; the differences between R3 and R2 are the effects of the δ^13^C_CH4-ruminant_ variation (i.e., the slightly decrease from −64.49‰ in 1961 to −64.93‰ in 2012). Source data are provided as a Source Data file

With our revised *F*_CH4-ruminant_ emissions and all other sources and sinks unchanged to their values from the baseline run, we simulate different trends in atmospheric CH_4_ compared with the baseline (Fig. 6a). During the period 1970–1989, our revised *F*_CH4-ruminant_ produces a slightly smaller simulated trend of CH_4_ (15.8 ppb yr^−1^) than the baseline (16.2 ppb yr^−1^). For the period 1990–1999, our estimate of *F*_CH4-ruminant_ (Table 2) produces similar trends of CH_4_ than the baseline. For the period after 2000, we obtain a larger trend of 4.1 ppb yr^−1^ compared with 3.0 ppb yr^−1^ in the baseline. This result suggests that our revised livestock emissions can explain a larger portion of the observed CH_4_ increase during 2000–2012, but that another source should be revised downwards by the same amount—or OH sink increased—to match with the observed CH_4_ trend.

Our revised *F*_CH4-ruminant_ (light blue line in Fig. 6c) alone, with δ^13^C_CH4-ruminant_ set the default value from ref. ^5,43^ (−62‰), implies a higher global isotopic source signature (δ^13^C_CH4-source_ weighted by all sources) due to our lower emissions than in EDGAR v4.3.2^10^. The δ^13^C_CH4-source_ differences with the baseline range from +0.11‰ in the 2000s to +0.18‰ in the 1970s. The smaller δ^13^C_CH4-source_ difference in the 2000s comes from the fact that the revised *F*_CH4-ruminant_ is closer to that of EDGAR v4.3.2^10^ during that period. Using our new δ^13^C_CH4-ruminant_ value shifts the global δ^13^C_CH4-source_ by −0.38‰ during 1980–2012 (difference between brown and light blue lines in Fig. 6c), which counterbalances the effect of our lower *F*_CH4-ruminant_. This result suggests that both *F*_CH4-ruminant_ and δ^13^C_CH4-ruminant_ revisions have significant impacts on δ^13^C_CH4-source_. Further studies using isotopic mass balances should thus not only include the revised source estimates for ruminants, but also revised δ^13^C_CH4-ruminant_.

Both the revised *F*_CH4-ruminant_ and δ^13^C_CH4-ruminant_ affect the mean values and trends of δ^13^C_CH4-atm_ in the box-model (Fig. 6b). From 1990 to 2012, compared with the baseline, the revised *F*_CH4-ruminant_ alone (light blue line in Fig. 6c) produces a larger mean δ^13^C_CH4-atm_ by +0.15‰. The revised δ^13^C_CH4-ruminant_ alone produces a lower mean value of δ^13^C_CH4-atm_ than the baseline by −0.37‰. The combined revised source and isotopic signature make the mean δ^13^C_CH4-atm_ smaller than the baseline by −0.22‰. However, the decreasing δ^13^C_CH4-ruminant_ has a very small effect on reconstructed δ^13^C_CH4-atm_ (by −0.02‰ only). This finding implies that even with substantial shifts in diet and geographical distribution on ruminant CH_4_ emissions, the temporal changes in δ^13^C_CH4-ruminant_ in the recent decades alone do not have significant effect on the reconstructed δ^13^C_CH4-atm_.

From 1990 to 2012, the box model prescribed with the revised *F*_CH4-ruminant_ simulates a smaller increase in δ^13^C_CH4-atm_ (change of +0.40‰ during 1990–2012; trend of +0.017‰ yr^−1^) compared with the baseline (change of +0.50‰; trend of +0.022‰ yr^−1^). Adding the new δ^13^C_CH4-ruminant_ makes the increase in δ^13^C_CH4-atm_ even smaller (change +0.31‰; trend of +0.013‰ yr^−1^). In other words, as compared with the baseline simulation using emissions from EDGAR v4.3.2^10^ and default δ^13^C_CH4-ruminant_ of −62‰, the updated *F*_CH4-ruminant_ and δ^13^C_CH4-ruminant_ from this study are responsible for a lower δ^13^C_CH4-atm_ by −0.19‰ between 1990 and 2012, and by −0.08‰ after 2006 when the CH_4_ growth rate became positive again^44,45^ . This corresponds to more than half of the observed decrease of δ ^13^C_CH4-atm_ (−0.15‰ between 2006 to 2012; derived from Table S4 of ref. ^5^). In conclusion, the box-model simulations have two main implications. Firstly, the revised CH_4_ emissions from ruminants have compensated δ^13^C_CH4-atm_ trend that could have otherwise increased more largely driven by the increasing fossil-fuel-related emissions. Secondly, the revised δ^13^C_CH4-ruminant_ to lower values gives a larger role of ruminant emissions in the recent δ^13^C_CH4-atm_ trend than previously estimated, consistent with the results from ref. ^5^.

## Methods

### Data

FAOSTAT—Live Animals and Livestock Primary^9^ provides annual national statistics on live animal stocks, numbers of slaughtered animal and laying poultry, milking animals (producing milk) and slaughtered animals (producing meat), and correspondent milk yield for milking animals (dairy cows, sheep, goats, and buffaloes for milk), meat yield (carcass weight) for meat animals (i.e., poultry, pigs, beef cattle, sheep, goats, and buffaloes for meat), and egg yield for laying poultry. In this study, poultry species include chickens, ducks, geese, turkeys, and other birds from FAOSTAT. Data of the period 1961–2012 were used in this study.

Concentrate feeds for livestock over the period 1961–2012 were derived from FAOSTAT—Commodity Balances^9^. In total, 74 feed commodities were presented in FAOSTAT (Supplementary Table 4). These feed commodities were regrouped into seven groups as those in ref. ^17^ (maize, other cereals, oilseeds, cakes of oilseeds, brans, pulses, and others (mainly starch crops and sugars); Supplementary Table 4) and their dry matter content calculated using dry matter to biomass ratios (Supplementary Table 5).

### Reconstructing the feedstuff for poultry and pigs

In this study, we adapted a simple feed model to determine the amount of concentrate feeds for poultry and pigs, and by difference to total feed commodities the amount that feeds ruminants. The details of the feed model and the methodology are described in Supplementary Note 1 (also see ref. ^17^). The input data for the feed model are concentrate feeds amounts, and animal stocks numbers and yields from FAOSTAT^9^. The 74 feed commodities (in dry matter) were grouped into seven groups of concentrate feeds categories: maize, other cereals, oilseeds, cakes of oilseeds, brans, pulses, and others (mainly starch crops and sugars; Supplementary Table 4). The supply of these concentrate feeds was distributed in priority to poultry and eggs producers, then to pigs according to their specific nutritional and energy demands^46^ established in the feed model (see Supplementary Note 1 for detail). Ruminant are assumed to receive all maize, other cereals, cakes of oilseeds, oilseeds, brans, and others feedstuff that are not consumed by poultry and pigs.

National- and time-dependent adjustments for farming intensity were applied to represent the situation that first, a share of the poultry and pig population are raised by smallholder farms as backyard production, and second, the fraction of backyard production in total production is decreasing in the recent decades following global intensification trend^18^. We assume that all smallholder farms backyard productions are based on local feed resources and did not use feed commodities included in FAOSTAT. In this study, the regional specific farming intensity in the year 2000 was derived from the fraction of smallholder production in literature (see Supporting information Sect. 4 of ref. ^18^). For developing countries, we assumed logistic increases in farming intensity during the period of 1960 and 2012, making the intensity value from literature reached by 2000 (Supplementary Fig. 2). The logistic increase is to mimic the intensification of pig and poultry production in developing countries characterized by a fast increase when intensification is low, and a slowing down at later stages when the level of intensification approaches the one of developed countries. An upper bound of 0.95 is set for farming intensity in developing countries, consistent with the maximum intensity indicated by Supporting information Sect. 4 of ref. ^18^. Farming intensities for developed countries were assumed to remain at the fraction of 2000 all the years through 1961 to 2012.

The original feed model was constructed based on an average nutrient and energy requirement of animals for a whole year and the animal stocks at the time of enumeration^9^. In this study, we adapted the feed composition (Supplementary Note 1), and calculated feed requirements using animal productions (slaughtered for meat and eggs) and feed conversion ratios (FCR), as given by:

6 $$Q_{n,i,j} = {\mathrm{Weight}}_{n,i,j} \times {\mathrm{FCR}}_n \times f_{{\mathrm{intensity}},i,j}$$

where *Q*_*n,i,j*_ is the total feed quantity (in kg dry matter) used for animal type *n* (poultry for meat, poultry for eggs, pigs for meat) in country *i* and year *j*; Weight_*n,i,j*_ is the total live weight of slaughtered animals (poultry and pigs for meat) and total weight of eggs in country *i* in year *j*; FCR_*n*_ defines the feed requirement in kg dry matter per kg body weight gain or per kg egg production for animal type *n* in year *i*; and *f*_intensity*,i,j*_ is the farming intensity in country *i* in year *j*.

During the recent decades, FCR of farm livestock keep decreasing due to the improvement in factors like nutrition and feeding practices (e.g., diet composition), health condition (reducing mortality), and killing weight (usually efficiency get worse after maturity). For developed countries, FCR = 1.95 kg dry matter (kg live-weight gain or kg eggs production)^−1^ in 2005 with a decreasing rate of −0.01 yr^−1^ for poultry live weight and eggs are used in this study. The FCR in 2005 is derived from the value for the United States Broiler Performance^47^, and the decreasing rate is assumed to generally fit the FCR evolution shown in the United States Broiler Performance. FCR = 3.28 kg dry matter (kg live weight gain)^−1^ in 1995 for pig live weight with a decreasing rate of −0.015 yr^−1^ are used in this study. The FCR in 1995 for pigs is derived from ref. ^48^ (compiled from statistics of the United States). The decreasing rate is roughly estimated using value of 1995 (3.28 kg dry matter (kg live-weight gain)^−1^) and of 2013 (just above 3 kg dry matter (kg live-weight gain)^−1^; see ref. ^49^), and assuming a linear change of the FCR. The FCRs of developing countries are assumed to be 20% higher than those of developed countries, given the example that China has 20% higher FCR for pigs than the United States^49^.

Weight_*n,i,j*_ for poultry for meat (Weight_poultry*,i,j*_), pigs (Weight_pig*,i,j*_), and eggs (Weight_egg*,i,j*_) in country *i* in year *j* are calculated as:

7 $${\mathrm{Weight}}_{{\mathrm{poultry}},i,j} = \frac{{{\sum} {N_{k,i,j} \times Y_{k,i,j}} }}{{f_{{\mathrm{dressing,poultry}}}}}$$

8 $${\mathrm{Weight}}_{{\mathrm{pig}},i,j} = \frac{{N_{{\mathrm{pig}},i,j} \times Y_{{\mathrm{pig}},i,j}}}{{f_{{\mathrm{dressing,pig}}}}}$$

9 $${\mathrm{Weight}}_{{\mathrm{egg}},i,j} = {\sum} {N_{{\mathrm{laying}},k,i,j} \times Y_{{\mathrm{egg}},k,i,j}}$$

where *N*_*k,i,j*_ and *Y*_*k,i,j*_ are the slaughtered numbers (in head) and the yield (in kg carcass weight per head) of poultry species *k* (chickens, ducks, geese, turkeys, and other birds) in country *i* in year *j*; *N*_pig*,i,j*_ and *Y*_pig*,i,j*_ are the slaughtered numbers (in head) and yield (in kg carcass weight per head) of pigs in country *i* in year *j*; *N*_laying_,_*k,i,j*_ and *Y*_egg*,k,i,j*_ are the laying numbers (in head) and yield (in kg eggs per head) of poultry species *k* (including hens and other birds) in country *i* in year *j*; *f*_dressing,poultry_ and *f*_dressing,pig_ are the dressing percentage of poultry and pig, respectively, representing the conversion factor between carcass weight and live weight. *f*_dressing,poultry_ = 70% and *f*_dressing,pig_ = 60%^50^ are used in this study.

### Reconstructing the feeds for ruminants

After having allocated concentrate feeds to poultry and pigs, ruminants are assumed to receive the remaining quantities of feed commodities (*Q*_concentrates_). In addition to feed commodities (concentrate feeds), ruminant livestock are mainly fed on grass but they also receive crop by-products (stover) and occasional feeds^18^. For grass-biomass consumed by ruminants (*Q*_grass_), we used the global livestock production dataset of gridded grass-biomass use for the year 2000 from ref. ^18^ extrapolated by ref. ^51^ backward and forward in time during 1961–2012 using metabolisable energy (ME) requirement of ruminants in each country. The rest of the consumption of biomass by ruminant livestock is assumed to be met by local stover and occasional feeds (hereafter, as other feeds), following ref. ^18^.

The quantities of other feeds in country *i* in year *j* (*Q*_*s+o,i,j*_) are calculated as the solution of the following equation:

10 $$\begin{array}{l}ME_{{\mathrm{ruminant}},i,j} = Q_{{\mathrm{s + o}},i,j} \times E_{{\mathrm{GE - feed}}} \times f_{{\mathrm{DE - s + o}}} \times REM_{{\mathrm{s + o}}}\\ + Q_{{\mathrm{concentrates}},i,j} \times E_{{\mathrm{GE - feed}}} \times f_{{\mathrm{DE - concentrates}}} \times REM_{{\mathrm{concentrates}}}\\ + Q_{{\mathrm{grass}},i,j} \times E_{{\mathrm{GE - feed}}} \times f_{{\mathrm{DE - grass}}} \times REM_{{\mathrm{grass}}}\end{array}$$

where *ME*_ruminant*,i,j*_ (MJ yr^−1^) is the total ME requirement of domestic ruminants in country *i* in year *j* derived from ref. ^51^; *E*_GE-feed_ is the average gross energy (GE) density of the feed with a value of 18.45 MJ kg^−1^ of dry matter as suggested by 2006 IPCC Guidelines for National Greenhouse Gas Inventories (ref. ^13^, Vol. 4, Chapter 10); *f*_DE-s+o_, *f*_DE-concentrates_, and *f*_DE-grass_ (in percent) are the digestible fractions of gross energy contained in other feeds, concentrate feeds, and grass-biomass, respectively. *f*_DE-concentrates_ is set to 80% (mean value) corresponding to the feed digestibility of concentrate diet (ref. ^13^, Vol. 4, Chapter 10, pp. 14, with a range of 75–85% as 95% confidence interval). *f*_DE-s+o_ and *f*_DE-grass_ are set to 55% (mean value) corresponding to the feed digestibility of medium quality forage (ref. ^13^, Vol. 4, Chapter 10, pp. 14, range of 45–65% as 95% confidence interval). *REM*_s+o_, *REM*_concentrates_, and *REM*_grass_ (in percent) are the fractions of digestible energy available in diet used for maintenance. REM parameter values themselves depend on *f*_DE_ and are calculated following 2006 IPCC Guidelines for National Greenhouse Gas Inventories (ref. ^13^, Vol. 4, Chapter 10, Eqn. 10.14).

### The isotopic signature of ruminant diet

The isotopic signature of ruminant diet in country *i* in year *j* (δ^13^C_diet*,i,j*_) is determined by the C3 and C4 fractions in ruminant diet, and can be calculated as:

11 $$\begin{array}{l}{\mathrm{\delta }}^{13}{\mathrm{C}}_{{\mathrm{diet}},i,j} = \frac{\sum Q_{{\mathrm{C3feed}},i,j} \times {\mathrm{\delta }}^{13}{\mathrm{C}}_{{\mathrm{C3feed}}} + \sum Q_{{\mathrm{C4feed}},i,j} \times {\mathrm{\delta }}^{13}{\mathrm{C}}_{{\mathrm{C4feed}}}}{\sum Q_{{\mathrm{C3feed}},i,j} + \sum Q_{{\mathrm{C4feed}},i,j}}\\ + \Delta _{{\mathrm{\delta }}^{13}{\mathrm{C}}_{{\mathrm{CO}}_{2} - {\mathrm{atm}},j}}\end{array}$$

where $Q_{{\mathrm{C3feed}},i,j}$ and $Q_{{\mathrm{C4feed}},i,j}$ are feed quantities (in kg dry matter) of C3-plant sources (including C3 concentrate feeds, C3 grasses, and C3 other feeds) and C4-plant sources (including C4 concentrate feeds, C4 grasses, and C4 other feeds) in country *i* in year *j*, respectively; ${\mathrm{\delta }}^{13}{\mathrm{C}}_{{\mathrm{C3feed}}}$ and ${\mathrm{\delta }}^{13}{\mathrm{C}}_{{\mathrm{C4feed}}}$ are the isotopic signature of different feeds for the year 2012; and $\Delta _{{\mathrm{\delta }}^{13}{\mathrm{C}}_{{\mathrm{CO}}_2 - {\mathrm{atm}},j}}$ is a factor to adjust the δ^13^C_diet_ to year *j* given the fact that the δ^13^C of plant synchronized decreases along δ^13^C_CO2-atm_ decrease^16^. $\Delta _{{\mathrm{\delta }}^{13}{\mathrm{C}}_{{\mathrm{CO}}_2 - {\mathrm{atm}},j}}$ is given by:

12 $$\Delta _{{\mathrm{\delta }}^{13}{\mathrm{C}}_{{\mathrm{CO}}_2 - {\mathrm{atm}},j}} = {\mathrm{\delta }}^{13}{\mathrm{C}}_{{\mathrm{CO}}_2 - {\mathrm{atm}},j} - {\mathrm{\delta }}^{13}{\mathrm{C}}_{{\mathrm{CO}}_2 - {\mathrm{atm}},2012}$$

where ${\mathrm{\delta }}^{13}{\mathrm{C}}_{{\mathrm{CO}}_2 - {\mathrm{atm}},j}$ and ${\mathrm{\delta }}^{13}{\mathrm{C}}_{{\mathrm{CO}}_2 - {\mathrm{atm}},2012}$ are isotopic signature of atmospheric CO_2_ for year *j* and the year 2012, respectively, which are derived from the observations of the Scripps CO_2_ Program (http://scrippsco2.ucsd.edu/) and compiled in ref. ^15^. Given the fact that the biomass used for feed are mainly the products of photosynthesis in the same year or growing season and subject to δ^13^C_CO2-atm_ of the year, we assumed no time-lag between the changes in δ^13^C_diet_ and that in δ^13^C_CO2-atm_. For ${\mathrm{\delta }}^{13}{\mathrm{C}}_{{\mathrm{C3feed}}}$ and ${\mathrm{\delta }}^{13}{\mathrm{C}}_{{\mathrm{C4feed}}}$, we used the following values derived from literature and adjusted to for the year 2012: −25.10 ± 2.27‰ for C3 concentrate feeds; −28.25 ± 1.68‰ for C3 grasses and other feeds; −12.24 ± 0.34‰ for C4 concentrate feeds (mainly maize); and −13.3 ± 1.1‰ for C4 grasses and other feeds (Supplementary Table 1).

Concentrate feeds (including all feed commodities left for ruminants and their C3 or C4 type), grasses (including C3 and C4 grasses), stover and occasional (including C3 and C4 other feeds) are major components of ruminants’ diet to satisfy their ME requirement^18^. Maize (*Q*_maize*,i,j*_), millet (*Q*_millet*,i,j*_), sorghum (*Q*_sorghum*,i,j*_), and sugarcane (*Q*_sugarcane*,i,j*_) are the major C4 concentrate feeds for ruminant:

13 $$Q_{{\mathrm{C4concentrates}},i,j} = Q_{{\mathrm{maize}},i,j} + Q_{{\mathrm{millet}},i,j} + Q_{{\mathrm{sorghum}},i,j} + Q_{{\mathrm{sugarcane}},i,j}$$

C3 concentrate feeds include concentrate feeds other than maize, millet, sorghum, and sugarcane:

14 $$\begin{array}{l}Q_{{\mathrm{C3concentrates}},i,j} = Q_{{\mathrm{concentrates}},i,j} - Q_{{\mathrm{maize}},i,j} - Q_{{\mathrm{millet}},i,j}\\ - Q_{{\mathrm{sorghum}},i,j} - Q_{{\mathrm{sugarcane}},i,j}\end{array}$$

where *Q*_concentrates*,i,j*_ is the remaining quantities of feed commodities after having allocated concentrate feeds to poultry and pigs. The quantities of millet (*Q*_millet,*i,j*_) and sorghum (*Q*_sorghum*,i,j*_) are part of other cereals category, and the quantities of sugarcane (*Q*_sugarcane*,i,j*_) are part of others category (Supplementary Table 4). In each country each year, the fractions of millet and sorghum in the other cereals category and sugarcane in the others category are assumed to keep the same values than in the initial feed commodities from FAOSTAT (i.e., before applying the simple feed model) and in the feeds left for ruminants (i.e., after applying the simple feed model). Components of ruminant diet other than concentrate feeds (i.e., grass and other feeds) are assumed to be produced and consumed locally. Thus we assumed the C3:C4 ratio of other feeds (i.e., *Q*_C3s+o,*i,j*_: *Q*_C4s+o*,i,j*_) the same as the ratio of grasses for each country (i.e., *Q*_C3grass*,i,j*_: *Q*_C4grass*,i,j*_) following a similar geographical C3 vs. C4 distribution^38^ (see below).

For grass-biomass consumed by ruminants, we used the global livestock production dataset of gridded grass-biomass use for the year 2000 from ref. ^18^ extrapolated by ref. ^51^ at the resolution of 0.5° × 0.5° backward and forward in time during 1961–2012 using ME requirement of ruminants in each country. To distinguish between C3 and C4 grass-biomass consumed, we used the (0.5° × 0.5°) maps prepared for the MsTMIP model intercomparison^52^ of the relative fraction of C3 and C4 grasses. The MsTMIP gridded C3–C4 grass distribution is derived from the approach described in ref. ^38^ based on growing season temperature (see Sect. 3.6 of ref. ^52^ for more details). Decadal maps of the relative fraction of C3 and C4 grasses were obtained using gridded CRU–NCEP mean monthly precipitation and temperature data^53^ from 1960s to 2000s. The relative fraction of C3 and C4 in each grid cell of the grass-biomass use were averaged at country level to set the C3 (*Q*_C3grass*,i,j*_) and C4 grass (*Q*_C4grass*,i,j*_) consumed by ruminants.

### Methane emissions from enteric fermentation of ruminants

Methane emission from enteric fermentation for country *i* in year *j* (*F*_CH4-ruminant,*i,j*_) is calculated using Eq. (15) adapted from IPCC Tier 2 algorithms (ref. ^13^, Vol. 4, Chapter 10, Eqn 10.21):

15 $$F_{{\mathrm{CH4 - ruminant}},i,j} = \frac{{\mathop {\sum }\nolimits GE_{{\mathrm{feed}},i,j} \times Y_{{\mathrm{m,feed}}}}}{{E_{{\mathrm{CH4}}} \times 10^9}}$$

where *GE*_ruminant*,i,j*_ (in MJ) is the annual gross energy intake by ruminants in country *i* in year *j*; *E*_CH4_ is the energy content of methane with value of 55.65 MJ (kg CH_4_)^−1^; *Y*_m,feed_ is the methane conversion factor expressed as the percent of gross energy in feed converted to methane; and 10^9^ is to convert the unit of methane emission from kg to Tg (i.e., 10^12^ g). In this study, *Y*_m_ = 6.5% ± 1.0% (the ± values represent the 95% confidence interval range) is used to represent wide-spread ruminant fed diets for cattle and mature sheep following IPCC guidelines^13^ (Vol. 4, Chapter 10, Tables 10.12 and 10.13), given the fact that globally, only little cattle in some developed countries is feedlot cattle with feed diets contain 90% or more concentrates. *GE*_feed*,i,j*_ is calculated as the gross energy content of different ruminant feeds (concentrates, grasses and other feeds):

16 $$GE_{{\mathrm{feed}},i,j} = Q_{{\mathrm{feed}},i,j} \times E_{{\mathrm{GE - feed}}}$$

### δ^13^C of diet and δ^13^C of enteric methane emissions

We collected published documents with δ^13^C_CH4-ruminant_ observations and, at the same time, with data on δ^13^C_diet_ or information of diet composition (quantities of specific feed categories or proportion of C3 and C4 feeds). Forty-three data from six published documents were collected^54–59^, and used to derive the relationship between δ^13^C_diet_ and δ^13^C_CH4-ruminant_. Within the 43 observations of δ^13^C_CH4-ruminant_, δ^13^C_diet_ of 27 observations are obtained directly from the documents. For the rest 16 observations, feed compositions are provided (Supplementary Table 2). We calculated the δ^13^C_diet_ as weighted average δ^13^C of feed compositions using the δ^13^C of different feed categories at the reference year 2012 (Supplementary Table 1), and adjusted the calculated δ^13^C_diet_ to the year when the δ^13^C_CH4-ruminant_ was measured (similar to Eqs. (11) and (12)). The δ^13^C of different feed categories were originally derived from literature with different years of sampling. To be comparable, they were first adjusted to the same year 2012 using the adjustment factor $\Delta _{{\mathrm{\delta }}^{13}{\mathrm{C}}_{{\mathrm{CO}}_2 - {\mathrm{atm}},j}}$ in Eq. (12). Mean value and uncertainty of the adjusted δ^13^C for each feed category was then calculated (Supplementary Table 1), and used to assess δ^13^C_diet_ and its uncertainty through Monte Carlo ensembles (*n* = 10,000; Supplementary Table 2).

The resulted relationship between δ^13^C_diet_ and δ^13^C_CH4-ruminant_ and their uncertainty (Fig. 2) are used to estimate the δ^13^C_CH4-ruminant_ of the global and national methane emissions from enteric fermentation (*F*_CH4-ruminant_) and its evolution since 1961.

### Uncertainty estimates

In this study, we assessed the uncertainties of the quantity of other feeds for ruminant (*Q*_s+o_; i.e., feeds other than concentrates and grasses; Eq. (10)), the δ^13^C of ruminant diet (δ^13^C_diet_; Eq. (11)), ruminant enteric methane emission (*F*_CH4-ruminant_; Eq. (15)), and its weighted δ^13^C (δ^13^C_CH4-ruminant_, using the fitted equation of δ^13^C_diet_ and δ^13^C_CH4-ruminant_ obtained in this study; Fig. 2). All the above uncertainties were assessed through Monte Carlo ensembles (*n* = 1000) considering the uncertainties of the parameters used in calculation. The information on the uncertainty assessment in this study is listed in Supplementary Table 3, including the resulting estimates, corresponding equations, and the parameters, and their uncertainties considered in the uncertainty assessment.

For the quantity of other feeds for ruminant (*Q*_s+o_), we assessed the uncertainties due to feed digestibility (parameters *f*_DE-s+o_, *f*_DE-concentrates_, and *f*_DE-grass_) and associated REMs (REM parameter values themselves dependent on *f*_DE_). For the δ^13^C of ruminant diet (δ^13^C_diet_), we assessed the uncertainties due to the *Q*_s+o_ and the δ^13^C of different feeds. For national and global methane emissions from enteric fermentation of ruminants (*F*_CH4-ruminant_), we assessed uncertainties due to the *Q*_s+o_ and the methane conversion factor (*Y*_m_). For the weighted δ^13^C_CH4_ of ruminants (δ^13^C_CH4-ruminant_), we assessed the uncertainties due to the uncertainties of δ^13^C_diet,*i,j*_ and the fitted linear regression between δ^13^C_diet_ and δ^13^C_CH4-ruminant_ constructed from observations.

### Description of the one-box model

The one-box model used in study is described in detail by ref. ^42,43^, and was recently used for studying methane sources from observed δ^13^C_CH4-atm_^5^. We commence our model integration at an imposed steady state in 1700 as in refs. ^42,43^, considering the methane concentration being fairly steady at near ca. 1700. As in ref. ^43^, we used bottom-up constructions of the methane source inventory (as inputs; see below), and then used the inferred sink history to close the budget for individual isotopologues (i.e., ^12^CH_4_ and ^13^CH_4_).

Global methane mass balance can be shown as:

17 $$\frac{{\partial C(t)}}{{\partial t}} = S\left( t \right) - \lambda \left( t \right)C(t)$$

where *S*(*t*), *λ*(*t*), and *C*(*t*) are the global methane source (Tg yr^−1^), sink (yr^−1^), and tropospheric burden (Tg), respectively, at time *t*. The model is performed at 1-year time step, thus ignoring seasonality. Assuming the source and sink are held constant within each time step, the analytic integration of Eq. (17) can be given by:

18 $$C_{{\mathrm{end}}} = \frac{S}{\lambda } + \left( {C_{{\mathrm{beg}}} - \frac{S}{\lambda }} \right)e^{ - \lambda \Delta t}$$

where the tropospheric burden (*C*(*t*); i.e., *C*_end_ and *C*_beg_) and source history (*S*(*t*)) are both specified at annual time steps. *C*(*t*) is proportional to global mean concentration (in ppb; see below on the inputs) with a conversion factor of 2.767 Tg ppb^−1 ^^60,61^. With bottom-up constructions of the methane source inventory (*S*(*t*) as inputs; Supplementary Fig. 3), the mass-balancing sink history (*λ*(*t*)) is deduced by solving Eq. (18) numerically in successive time steps of *λ* (Supplementary Fig. 3c). With the usual assumption of *λ*_13_ = *αλ*_12_, where *α* is the sink weighted fractionation factor given by *ε* = *α* − 1 = −6.9‰^5^, we could deduce *λ*_13_(*t*) and *λ*_12_(*t*) separately from *λ*(*t*) by imposing mass balance for total methane and for each methane isotopologue (with *S*(*t*) and its isotopologue components *S*_13_(*t*) and *S*_12_(*t*) as inputs; Sect. 4 of ref. ^43^). *S*_13_(*t*) and *S*_12_(*t*) are calculated from *S*(*t*) for each inventory component using a representative δ^13^C from Table 1 of ref. ^43^ (e.g., −62‰ for livestock enteric emissions; Supplementary Table 6).

To quantify the effects of the revised *F*_CH4-ruminant_ and δ^13^C_CH4-ruminant_ individually or together on atmospheric CH_4_ concentration and δ^13^C_CH4-atm_, the model was run in forward mode (see below for the details of the simulations). A baseline simulation started in 1700, using the bottom-up constructions of the methane source inventory (*S*_13_(*t*) and *S*_12_(*t*)) and the deduced mass-balancing sink history (*λ*_13_(*t*) and *λ*_12_(*t*)) for each methane isotopologue over the historical period (1700–2012). R1 simulation implemented a perturbation in the baseline that commences in 1960 with the revised *F*_CH4-ruminant_ and kept the rest sources identical to the baseline simulation (e.g., δ^13^C_CH4-ruminant_ set to default value of −62‰ previously used by ref. ^5,43^). In R2 simulation, the revised *F*_CH4-ruminant_ and δ^13^C_CH4-ruminant_ (including the slightly decrease from −64.49‰ in 1961 to −64.93‰ in 2012) were used. For the spin-up period of 1700–1960 in R2 simulation, the revised δ^13^C_CH4-ruminant_ of 1961 (−64.49‰) was used. To run the R2 simulation, we first re-calculated *S*_13_(*t*) and *S*_12_(*t*) using revised δ^13^C_CH4-ruminant_, and re-deduced *λ*_13_(*t*) and *λ*_12_(*t*) as inputs. The R3 simulation is the same as R2 but with constant δ^13^C_CH4-ruminant_ at −64.49‰ for the period of 1961–2012. Therefore, the resulted differences between R1 and baseline are the effects of the revised *F*_CH4-ruminant_; the differences between R2 and R1 are the effects of the revised δ^13^C_CH4-ruminant_; and the differences between R3 and R2 are the effects of the δ^13^C_CH4-ruminant_ variation (i.e., the slightly decrease from −64.49‰ in 1961 to −64.93‰ in 2012). It should be noted that the purpose of the simulations is not to reproduce the observed δ^13^C_CH4-atm_, but to get a reasonable δ^13^C_CH4-atm_ in 1960, and the purpose of the perturbed simulations R1, R2, and R3 is to test the effects of the *F*_CH4-ruminant_ and δ^13^C_CH4-ruminant_ revisions on the post-1960 trends from an unperturbed 1960.

In the original box model, the mass-balancing sink (*λ*(*t*)) during the historical period (1700–2012) was tuned to match exactly atmospheric observations (Supplementary Fig. 3c). Here, we want to illustrate the effect of changing ruminant emission and its ^13^C source signature, and the results from our sensitivity tests thus deviate from atmospheric observations. A re-tuning of the model to match observations of mean concentration (with our revised *F*_CH4-ruminant_ for the period of 1961–2012) is beyond the scope of this study, but would only require a change in the global mass-balancing sink of 2.4% during 1961–2012 (from 4.9% in 1961 to <1% after 2010; see difference between red and black lines in Supplementary Figure 3c), which is well within the uncertainty of the atmospheric sink. An additional sensitivity test shows that the re-tuning of the global mass-balancing sink only has marginal effect on the results presented in the Discussion section.

Inputs for the box model include the global methane concentration (Supplementary Fig. 3), and the construction of the methane source inventory from 1700 (Supplementary Table 6). The global methane concentration since 1984 is derived from observations of a globally distributed network (NOAA-ESRL record^44,62^). Data for 1700–1983 are from ref. ^63^, converted to the NOAA04 scale^64^ and adjusted by 45% of the inter-polar gradient to adjust Antarctic to global values^5^.

For the construction of the methane source inventory, we combined the natural and anthropogenic emissions of 1700 derived from Table 1 of ref. ^43^, the historical anthropogenic emissions^65^ (1850–2000) used by the Atmospheric Chemistry and Climate Model Intercomparison Project (ACCMIP), and the EDGAR v4.3.2^10^ for the recent decades (Supplementary Table 6). Five natural emission sources are used in this study following Table 1 of ref. ^43^: wetlands, termites, oceans, wild animals, and geologic sources. We assumed that the natural emissions are kept constant across history. Nine anthropogenic sources are used including fossil fuel (industry), waste treatment and landfill, rice cultivation, livestock enteric fermentation, manure management, and four pyrogenic sources (agricultural waste burning, forest burning, C3 grass burning, and savanna (C4 grass) burning). The emissions of 1700 are derived from Table 1 of ref. ^43^ except pyrogenic sources (see below). Reference ^65^ provides gridded (0.5° × 0.5°) historical anthropogenic methane emissions from 1850 to 2000. This dataset includes fossil fuel, waste treatment and landfill, and different pyrogenic sources (agricultural waste burning, forest burning, and grass burning), but gathers rice cultivation, livestock enteric fermentation, manure management as a single agricultural sector source. To separate grass burning into C3 grass burning and savanna (C4 grass) burning, we applied the gridded C3–C4 grass distribution derived from the approach described in ref. ^38^ based on growing season temperature. The EDGAR v4.3.2^10^ provides emissions from fossil fuel, waste treatment and landfill, rice cultivation, livestock enteric fermentation, manure management, and agricultural waste burning for the period of 1970–2012. To separate the agricultural sector emissions from ref. ^65^ into different components (for the period of 1850–1970), we applied the relative fractions of rice cultivation, livestock enteric fermentation, and manure management from EDGAR v4.3.2^10^ for the year 1970. For pyrogenic emissions, we did not use fire emissions (wildfires, forest burning and savanna burning) of 1700 in the Table 1 of ref. ^43^. Instead, we used pyrogenic emissions of ref. ^65^, and assumed that they are constant between 1700 and 1850. For each of the anthropogenic source, we applied linear interpolation between 1700 (Table 1 of ref. ^43^) and 1850^65^, and between 1850 and 1970 (for each decade) to obtain annual emissions. For the year since 1970, values from the EDGAR v4.3.2^10^ were used.

## Supplementary information

Supplementary Information

Source Data

## Data Availability

The data that support the findings of this study are available at the public Data Repository of the International Institute of Applied Systems Analysis (IIASA DARE; 10.22022/ESM/06-2019.45). The source data underlying Figs. 1, 3, and 6 are provided as a Source Data file. The source data underlying Fig. 2 are provided by Supplementary Table 2. The source data underlying Figs. 4 and 5 can be found in the IIASA DARE (10.22022/ESM/06-2019.45).
