# Supplementary material for: Revisiting enteric methane emissions from domestic ruminants and their δ13CCH4 source signature
Source: Nat Commun. 2019 Jul 31;10:3420. doi: 10.1038/s41467-019-11066-3 (PMC6668425; doi:10.1038/s41467-019-11066-3)
Supplement: Supplementary file 1 — Supplementary Information [file 41467_2019_11066_MOESM1_ESM.docx]

**Supplementary information for**

**Revisiting enteric methane emissions from domestic ruminants and their *δ^13^C_CH4_* source signature**

Chang et al.


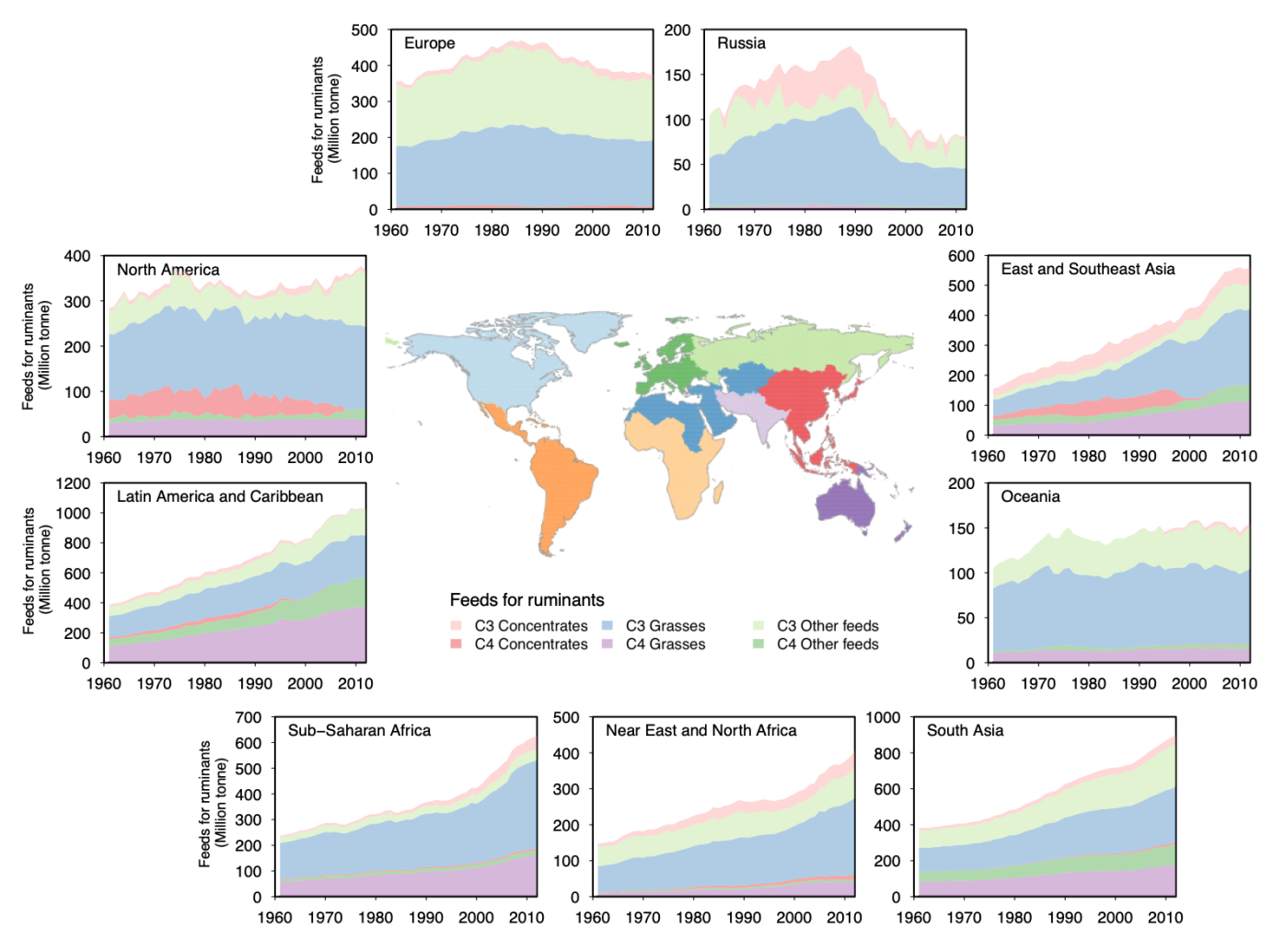


**Supplementary Figure 1. The regional changes in the composition of feeds for ruminants over the period of 1961-2012.** The feeds for ruminants are concentrates (including all crop grains feed commodities for ruminants, C3-based or C4-based), grass (including C3 and C4 grasses), and other feeds (i.e., stover and occasional feeds including C3 and C4 part) following ref ^1^. The mean amount of C3 and C4 other feeds consumed by ruminants estimated in this study is used in the stacked plots.

**Supplementary Figure 2. Examples of the logistic increase of feeding intensity in developing countries in this study.** A shape of a standard logistic function is used here (i.e., *y = 1/(1+exp(x))*). For a given farming intensity in 2000 (derived from Supporting information Sect. 4 of ref ^1^), we assumed logistic increases in farming intensity during the period of 1960 and 2012, with the given farming intensity reached by 2000. An upper bound of 0.95 is set for farming intensity in developing countries, which is the maximum intensity indicated by Supporting information Sect. 4 of ref ^1^.


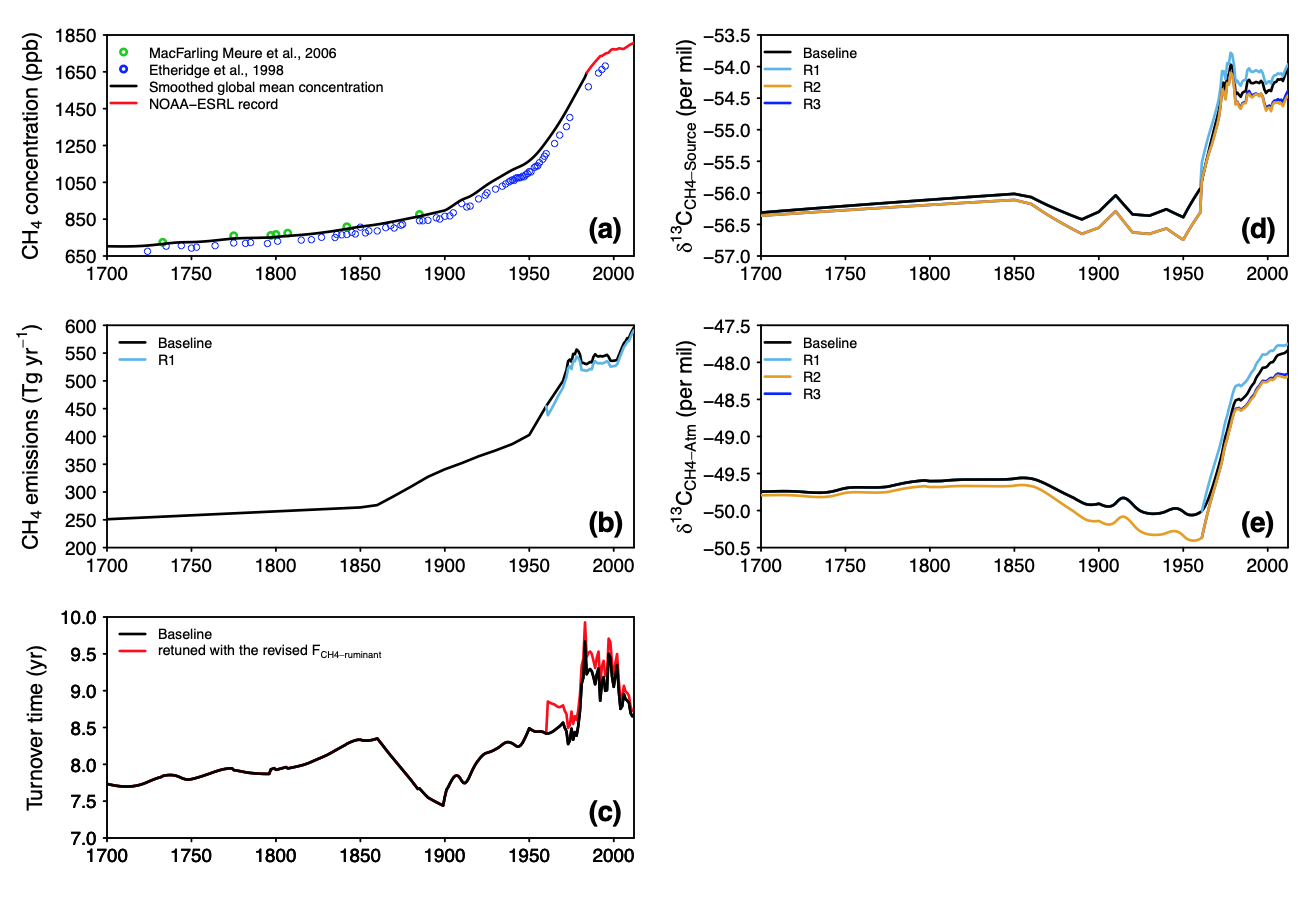


**Supplementary Figure 3.** **(a) The global atmospheric methane concentration, (b) the construction of the methane emission history, (c) the histories of methane turnover time corresponding to the construction of the methane emission history, (d) the isotopic source signature weighted by all sources (*δ^13^C_CH4-source_*), and (e) the box model results on *δ^13^C_CH4-atm_*.** In the simulation of R1, only the revised *F_CH4-ruminant_* is used and *δ^13^C_CH4-ruminant_* set to default value of -62‰ previously used by ref ^2,3^. In R2, the revised *F_CH4-ruminant_* and *δ^13^C_CH4-ruminant_* are used (see Methods). R3 is the same as R2 but with constant *δ^13^C_CH4-ruminant_* at -64.49‰ for the period of 1961-2012. The differences between R1 and baseline are the effects of the revised *F_CH4-ruminant_*; the differences between R2 and R1 are the effects of the revised *δ^13^C_CH4-ruminant_*; the differences between R3 and R2 are the effects of the *δ^13^C_CH4-ruminant_* variation (i.e., the slightly decrease from -64.49‰ in 1961 to -64.93‰ in 2012).

**Supplementary Table 1. *δ^13^C* of different feed categories derived from literature.**

| Feed category | Species / subcategory | Year of sampling | *δ^13^C_feed_* (‰) | Adjusted *δ^13^C_feed_* at 2012 (‰) | Reference |
| --- | --- | --- | --- | --- | --- |
| Alfalfa | Alfalfa | 1997 | -27.6 | -27.98 | González-Martin et al., 1999 ^4^ |
|  | Alfalfa | 1997 | -27.401 | -27.781 | González-Martin et al., 1999^4^ |
| Soybeans (Soya) | Soya | 1997 | -25.416 | -25.796 | González-Martin et al., 1999^4^ |
|  | Soy grist | 2005 | -25.6 ± 0.3 | -25.75 ± 0.3 | Knobbe et al., 2006^5^ |
|  | Soybean meal | 2004 | -25.1 | -25.27 | Camin et al., 2008^6^ |
| Barley | Barley | 1997 | -22.148 | -22.528 | González-Martin et al., 1999^4^ |
|  | Barley | 1987 | -25.3 | -25.91 | Wilson et al., 1988^7^ |
|  | Barley | 2005 | -26.7 | -26.85 | Camin et al., 2008^6^ |
| Wheat | Wheat | 1997 | -22.845 | -23.225 | González-Martin et al., 1999^4^ |
| Maize (Corn) | Corn | 1997 | -11.183 | -11.563 | González-Martin et al., 1999^4^ |
|  | Maize silage | 1987 | -11.7 | -12.31 | Wilson et al., 1988^7^ |
|  | Maize meal | 1987 | -11.6 | -12.21 | Wilson et al., 1988^7^ |
|  | Maize silage | 2000 | -11.9 ± 0.4 | -12.2 ± 0.4 | De Smet et al., 2004^8^ |
|  | Maize | 2004 | -12.5 ± 0.1 | -12.67 ± 0.1 | Knobbe et al., 2006^5^ |
|  | Maize silage | 2005 | -12.4 | -12.55 | Camin et al., 2008^6^ |
|  | Maize silage | 2005 | -12.5 | -12.65 | Camin et al., 2008^6^ |
|  | Maize flour | 2005 | -11.8 | -11.95 | Camin et al., 2008^6^ |
|  | Maize silage | 2005 | -12.1 | -12.25 | Camin et al., 2008^6^ |
|  | Maize flour | 2005 | -11.9 | -12.05 | Camin et al., 2008^6^ |
| Forages - C3 plants | Spring pasture | 1987 | -29.3 | -29.91 | Wilson et al., 1988^7^ |
|  | Autumn pasture | 1987 | -28.6 | -29.21 | Wilson et al., 1988^7^ |
|  | Grass | 2000 | -29.8 ± 0.2 | -30.1 ± 0.2 | De Smet et al., 2004^8^ |
|  | Triticale silage | 2000 | -27.3 ± 1.9 | -27.6 ± 1.9 | De Smet et al., 2004^8^ |
|  | Grass silage | 2000 | -29.0 ± 1.2 | -29.3 ± 1.2 | De Smet et al., 2004^8^ |
|  | Straw | 2000 | -29.3 ± 0.1 | -29.6 ± 0.1 | De Smet et al., 2004^8^ |
|  | Grass | 2004 | -27.9 ± 0.4 | -28.07 ± 0.4 | Knobbe et al., 2006^5^ |
|  | Hay | 2004 | -28.1 ± 0.9 | -28.27 ± 0.9 | Knobbe et al., 2006^5^ |
|  | Straw | 2004 | -26.9 ± 0.3 | -27.07 ± 0.3 | Knobbe et al., 2006^5^ |
|  | Medicago sativa | 2005 | -28.7 | -28.85 | Camin et al., 2008^6^ |
|  | Medicago sativa | 2005 | -27.3 | -27.45 | Camin et al., 2008^6^ |
|  | Festuca arudinacea | 2005 | -23.2 | -23.35 | Camin et al., 2008^6^ |
|  | Festuca arudinacea | 2005 | -25.3 | -25.45 | Camin et al., 2008^6^ |
|  | mixture of Medicago & Festuca | 2005 | -25.7 | -25.85 | Camin et al., 2008^6^ |
|  | mixture of Medicago & Festuca | 2005 | -28.3 | -28.45 | Camin et al., 2008^6^ |
|  | Medicago sativa | 2005 | -28.4 | -28.55 | Camin et al., 2008^6^ |
|  | Medicago sativa | 2005 | -28.8 | -28.95 | Camin et al., 2008^6^ |
|  | Lolium multiflorum | 2005 | -29.5 | -29.65 | Camin et al., 2008^6^ |
|  | Lolium multiflorum | 2005 | -29.6 | -29.75 | Camin et al., 2008^6^ |
|  | mixture of Medicago & Lolium | 2005 | -28.8 | -28.95 | Camin et al., 2008^6^ |
|  | mixture of Medicago & Lolium | 2005 | -28.8 | -28.95 | Camin et al., 2008^6^ |
| ***δ^13^C_feed_* of each feed category synthesized from values above (used to calculate *δ^13^C_diet_* in Supplementary Table 2)** | | | | | |
| Alfalfa |  | 2012 |  | -27.88 ± 0.14 |  |
| Soybeans |  | 2012 |  | -25.60 ± 0.29 |  |
| Maize |  | 2012 |  | -12.24 ± 0.34 |  |
| Barley |  | 2012 |  | -25.10 ± 2.27 |  |
| Wheat |  | 2012 |  | -23.61 |  |
| Forages - C3 plants |  | 2012 |  | -28.25 ± 1.68 |  |
| ***δ^13^C_feed_* from other literature** | | | | | |
| C3 plants |  | ca. 1970s* | -27.1 ± 2.0 | -28.0 ± 2.0 | O'Leary, 1988^9^ |
| C4 plants |  | ca. 1970s* | -13 ± 1.2 | -13.9 ± 1.2 | O'Leary, 1988^9^ |
| Modern C3 grasses |  | ca. 1970s-1980s# | -26.7 ± 2.3 | -27.5 ± 2.3 | Cerling et al., 1997^10^ |
| Modern C4 grasses |  | ca. 1970s-1980s# | -12.5 ± 1.1 | -13.3 ± 1.1 | Cerling et al., 1997^10^ |
| ***δ^13^C_feed_* used to calculate *δ^13^C_diet_* for each country (see Methods)** | | | | | |
| C3 grasses and other feeds | |  |  | -28.25 ± 1.68 |  |
| C4 grasses and other feeds | |  |  | -13.3 ± 1.1 |  |
| C3 concentrate feeds | |  |  | -25.10 ± 2.27 |  |
| C4 concentrate feeds | |  |  | -12.24 ± 0.34 |  |

**Supplementary Table 2. *δ^13^C* of diet (*δ^13^C_diet_*) and the corresponding *δ^13^C* of ruminant CH_4_ emissions *δ^13^C_CH4-ruminant_* derived from literature.**

| Livestock category | Year of sampling | Composition of feeds | *δ^13^C_diet_* (‰) | *δ^13^C_CH4-ruminant_* (‰) | Reference |
| --- | --- | --- | --- | --- | --- |
| Cow | 1979 | Timothy and bromegrasses | -27.45 ± 1.68* | -61.60 | Rust, 1981^11^ |
| Steer | 1979 | Alfalfa (13 lb/day), ground soybeans plus corn (4 lb/day), and corn silage (4 lb/day) | -22.40 ± 0.21* | -61.80 | Rust, 1981^11^ |
| Steer | 1979 | Alfalfa | -27.08 ± 0.14* | -61.10 | Rust, 1981^11^ |
| Steer | 1980 | Alfalfa (15 lb/day), ground corn (4 lb/day), and soybeans (1 lb/day) | -25.43 ± 0.18* | -58.10 | Rust, 1981^11^ |
| Steer | 1980 | Alfalfa (15 lb/day), ground corn (4 lb/day), and soybeans (1 lb/day) | -25.43 ± 0.18* | -76.00 | Rust, 1981^11^ |
| Dairy herd of 40 animals | 1980 | Alfalfa | -27.10 ± 0.14* | -61.10 | Rust, 1981^11^ |
| Wether | 1980 | Alfalfa | -27.10 ± 0.14* | -70.20 | Rust, 1981^11^ |
| Wether | 1980 | Alfalfa | -27.10 ± 0.14* | -67.00 | Rust, 1981^11^ |
| Steer | 1979 | Corn silage (10 lb/day), and ground corn and soybeans (14 lb/day) | -15.34 ± 0.33* | -50.60 | Rust, 1981^11^ |
| Cow | 1980 | Corn silage (32 lb/day), ground corn (36.8 lb/day), soybeans (9.2 lb/day) | -13.04 ± 0.34* | -52.80 | Rust, 1981^11^ |
| Cow | 1980 | Corn silage (30 lb/day), ground corn (32 lb/day), soybeans (8 lb/day) | -12.99 ± 0.34* | -47.40 | Rust, 1981^11^ |
| Beef herd of 300 animals | 1980 | Mostly corn (a C4 plant)† | -13.06 ± 1.04* | -45.40 | Rust, 1981^11^ |
| Cow | ca. 1988# | 100% C3 diet | -27.44 ± 1.68* | -65.10 | Levin et al., 1993^12^ |
| Cow | ca. 1988# | 60-80% C4 diet | -16.24 ± 1.19* | -55.60 | Levin et al., 1993^12^ |
| Sheep | ca. 1988# | C3 diet† | -27.44 ± 1.68* | -70.60 | Levin et al., 1993^12^ |
| Goat | ca. 1988# | C3 diet† | -27.44 ± 1.68* | -65.20 | Levin et al., 1993^12^ |
| Cow | 1995 | 60% ryegrass hay, 40% concentrate | -25.60 | -67.77¶ | Bilek et al., 2001^13^ |
| Cow | 1995 | 60% ryegrass hay, 40% concentrate | -25.60 | -67.80¶ | Bilek et al., 2001^13^ |
| Cow | 1995 | 80% ryegrass hay, 20% concentrate | -27.60 | -69.80¶ | Bilek et al., 2001^13^ |
| Cow | 1995 | 40% ryegrass hay, 60% concentrate | -23.70 | -63.69¶ | Bilek et al., 2001^13^ |
| Cow | 1996 | 50% orchard ryegrass hay, 25% Italian ryegrass hay, 25% perennial ryegrass hay | -28.90 | -74.91¶ | Bilek et al., 2001^13^ |
| Cow | 1996 | 50% orchard ryegrass hay, 25% Italian ryegrass hay, 25% perennial ryegrass hay | -28.90 | -74.58¶ | Bilek et al., 2001^13^ |
| Cow | ca. 2008§ | Maize | -15.20 | -65.50 | Klevenhusen et al., 2009^14^ |
| Cow | ca. 2008§ | Maize and wheat | -21.20 | -68.30 | Klevenhusen et al., 2009^14^ |
| Cow | ca. 2008§ | Wheat | -28.70 | -73.40 | Klevenhusen et al., 2009^14^ |
| Cow | ca. 2008§ | Hay | -30.40 | -66.40 | Klevenhusen et al., 2009^14^ |
| Cow | ca. 2008§ | Maize + 50g/kg DM Monolaurin | -16.90 | -67.40 | Klevenhusen et al., 2009^14^ |
| Cow | ca. 2008§ | Maize and wheat + 50g/kg DM Monolaurin | -23.30 | -69.10 | Klevenhusen et al., 2009^14^ |
| Cow | ca. 2008§ | Wheat + 50g/kg DM Monolaurin | -28.60 | -69.60 | Klevenhusen et al., 2009^14^ |
| Cow | ca. 2008§ | Hay + 50g/kg DM Monolaurin | -30.30 | -67.10 | Klevenhusen et al., 2009^14^ |
| Cow | ca. 2009§ | Hay | -26.00 | -67.20 | Klevenhusen et al., 2010^15^ |
| Cow | ca. 2009§ | Barley and soybean meal | -25.70 | -67.90 | Klevenhusen et al., 2010^15^ |
| Cow | ca. 2009§ | Maize | -13.50 | -57.50 | Klevenhusen et al., 2010^15^ |
| Cow | ca. 1988 | Beet sucrose | -25.9 | -62.3 | Metges et al., 1990 ^16^ |
| Cow | ca. 1988 | Dried sugar-beet pulp | -26.9 | -75.6 | Metges et al., 1990^16^ |
| Cow | ca. 1988 | Dried sugar-beet pulp | -26.9 | -70.6 | Metges et al., 1990^16^ |
| Cow | ca. 1988 | Hay | -28.5 | -72.1 | Metges et al., 1990^16^ |
| Cow | ca. 1988 | C3 diet | -27.1 ± 1.1 | -70.2 ± 5.6 | Metges et al., 1990^16^ |
| Cow | ca. 1988 | Maize glucose | -10.7 | -56 | Metges et al., 1990^16^ |
| Cow | ca. 1988 | Ground maize | -12.2 | -55.5 | Metges et al., 1990^16^ |
| Cow | ca. 1988 | Ground maize | -12.2 | -52.9 | Metges et al., 1990^16^ |
| Cow | ca. 1988 | Maize cob husks | -12.9 | -60.5 | Metges et al., 1990^16^ |
| Cow | ca. 1988 | C4 diet | -12.0 ± 0.9 | -56.2 ± 3.2 | Metges et al., 1990^16^ |

* For *δ^13^C_diet_* that are not measured directly, we used the *δ^13^C* of different C3 and C4 feed components calculated from the values in Supplementary Table 1 (mean ± standard deviation for the year 2012). For diet with mixture of different components, a Monte Carlo simulation (n = 1000) was used to calculate the *δ^13^C* of mixed diet. For "ground corn and soybeans", we assumed that each of them comprises half of the meal. To account for the impact of decreasing *δ^13^C_CO2-atm_* on *δ^13^C* of feeds, we adjusted the calculated *δ^13^C_diet_* to the year when the *δ^13^C_CH4-ruminant_* was measured.

¶ We used engauge-digitizer (<https://github.com/markummitchell/engauge-digitizer/releases)> to digitize the data from Figure 2 in Bilek et al. (2001) ^13^ excluding their Experiment 4 that do not have enough samples as the other experiments.

† For diet of “Mostly corn”, 80%-100% maize with rest 0-20% of forages - C3 plants is assumed. For diet of “C3 diet”, 100% of forages - C3 plants is assumed.

# The exact sample collection date for ruminant emissions is not presented in the study. We used the collection year for soil gas sampling in this study.

§ The exact sample collection date for ruminant emissions is not presented in the study. We used the year when the paper was submitted.

**Supplementary Table 3. Uncertainty assessment in this study.**

| Estimates | Equation | Methods of uncertainty assessment | Parameters | Mean value and uncertainty of parameters | Source |
| --- | --- | --- | --- | --- | --- |
| *Q_n,i,j_* | (6) | No uncertainty is assessed | *Weight_n,i,j_* |  | Equation (7-9) |
|  |  |  | *FCR_n_* |  | Losinger, 1998^17^; National Chicken Council, 2018^18^; Rabobank Research, 2015^19^ |
|  |  |  | *f_intensity,i,j_* |  | See Methods and Supplementary Figure 2 |
| *Weight_n,i,j_* | (7-9) | No uncertainty is assessed | *N_k,i,j_*, *N_pig,i,j_*, and *N_laying_,_k,i,j_* |  | FAOSTAT^20^ |
|  |  |  | *Y_k,i,j_*, *Y_pig,i,j_*, and *Y_egg,k,i,j_* |  | FAOSTAT^20^ |
|  |  |  | *f_dressing,poultry_* and *f_dressing,pig_* | *f_dressing,poultry_* = 70%  *f_dressing,pig_* = 60% | Verheijen et al., 1996^21^ |
| *Q_s+o,i,j_* | (10) | Uncertainty is assessed through Monte Carlo ensemble (n = 1,000) accounting for the uncertainties of  *f_DE-concentrates_*, *f_DE-grass_*, *f_DE-s+o_*, *REM_concentrates_*, *REM_grass_*, and *REM_s+o_* | *ME_ruminant,i,j_* |  | Herrero et al., 2013^1^; Chang et al., 2016^22^ |
|  |  |  | *Q_concentrates,i,j_* | concentrate feeds for ruminant as the difference between total available concentrate feeds from FAOSTAT and those consumed by pigs and poultry (i.e., *Q_n,i,j_*) | Equation (6); see Methods |
|  |  |  | *Q_grass,i,j_* |  | Herrero et al., 2013^1^; Chang et al., 2016^22^ |
|  |  |  | *E_GE-feed_* | 18.45 MJ kg^-1^ of dry matter | IPCC, 2006 (Vol 4, Chapter 10) ^23^ |
|  |  |  | *f_DE-concentrates_*, *f_DE-grass_* and *f_DE-s+o_* | 80% [75% - 85% as 95 percent confidence interval] for *f_DE-concentrates_*, and 55% [45% - 65% as 95 percent confidence interval] for *f_DE-grass_* and *f_DE-s+o_* | IPCC, 2006 (Vol 4, Chapter 10) ^23^ |
|  |  |  | *REM_concentrates_*, *REM_grass_* and *REM_s+o_* | Values are calculated based on *f_DE_*, so as the uncertainties | IPCC, 2006 (Vol 4, Chapter 10, Eqn. 10.14) ^23^ |
| *δ^13^C_diet,i,j_* | (11) | Uncertainty is assessed through Monte Carlo ensemble (n = 1,000) accounting for the uncertainties of  *Q_C3s+o,i,j_*, *Q_C4s+o,i,j_*,  *δ^13^C_C3concentrates_*, *δ^13^C_C4concentrates_*, *δ^13^C_C3grass_*, *δ^13^C_C4grass_*, *δ^13^C_C3s+o_*, and *δ^13^C_C4s+o_* | *Q_C3concentrates,i,j_* and *Q_C4concentrates,i,j_* | C3 and C4 component is specified in *Q_concentrates,i,j_* | See Methods |
|  |  |  | *Q_C3grass,i,j_* and *Q_C4grass,i,j_* | Derived from $Q_{grass,i,j}$ with separation of C3 and C4 component | Herrero et al., 2013^1^; Chang et al., 2016^22^; the gridded C3-C4 fraction is derived from the approach described in Still et al. (2003) ^24^ based on growing season temperature. |
|  |  |  | *Q_C3s+o,i,j_* and *Q_C4s+o,i,j_* | Mean value and uncertainty is assessed in Equation (5) with separation of C3 and C4 component | Equation (10); The gridded C3-C4 fraction is the same as that of grasses in each country at each year. |
|  |  |  | *δ^13^C_C3concentrates_* and *δ^13^C_C4concentrates_* | *δ^13^C_C3concentrates_* = -25.10 ± 2.27‰  *δ^13^C_C4concentrates_* = -12.24 ± 0.34‰ | Literature; see Supplementary Table 1 |
|  |  |  | *δ^13^C_C3grass_* and *δ^13^C_C4grass_* | *δ^13^C_C3grass_* = -28.25 ± 1.68‰  *δ^13^C_C4grass_* = -13.3 ± 1.1‰ | Literature; see Supplementary Table 1 |
|  |  |  | *δ^13^C_C3s+o_* and *δ^13^C_C4s+o_* | The same as *δ^13^C_C3grass_* and *δ^13^C_C4grass_* respectively | Literature; see Supplementary Table 1 |
|  |  |  | $\Delta_{{\delta^{13}C}_{{CO}_{2}-atm,j}}$ |  | Equation (12) |
| $\Delta_{{\delta^{13}C}_{{CO}_{2}-atm,j}}$ | (12) | No uncertainty is assessed | *δ^13^C_CO2-atm,j_* and *δ^13^C_CO2-atm,2012_* |  | The Scripps CO_2_ Program (<http://scrippsco2.ucsd.edu/)>; data compiled in Graven et al., 2017 |
| *F_CH4-ruminant,i,j_* | (15) | Uncertainty is assessed through Monte Carlo ensemble (n = 1,000) accounting for the uncertainties of *Y_m,feed_* and *GE_feed,i,j_* | *GE_feed,i,j_* | Mean value and uncertainty of *GE_feed,i,j_* is assessed in Equation (16) | Equation (16) |
|  |  |  | *Y_m,feed_* (i.e., *Y_m,concentrates_*, *Y_m,grass_*, and *Y_m,s+o_*) | *Y_m_* = 6.5% ± 1.0% (the ± values represent the 95 percent confidence interval range) | IPCC, 2006 (Vol 4, Chapter 10, Table 10.12 and 10.13) ^23^ |
|  |  |  | *E_CH4_* | 55.65 MJ (kg CH_4_)^-1^ | IPCC, 2006 (Vol 4, Chapter 10) ^23^ |
| *GE_feed,i,j_* | (16) | Uncertainty is assessed through Monte Carlo ensemble (n = 1,000) accounting for the uncertainty of *Q_s+o,i,j_* | *Q_concentrates,i,j_*, *Q_grass,i,j_*, and *Q_o+s,i,j_* | Mean value and uncertainty of *Q_o+s,i,j_* is assessed in Equation (10) | Equation (510; see above |
|  |  |  | *E_GE-feed_* | 18.45 MJ kg^-1^ of dry matter | IPCC, 2006 (Vol 4, Chapter 10) ^23^ |
| *δ^13^C_CH4-ruminant,i,j_* |  | Uncertainty is assessed through Monte Carlo ensemble (n = 1,000) accounting for the uncertainty of  *δ^13^C_diet,i,j_* and the linear regression between *δ^13^C_diet_* and *δ^13^C_CH4-ruminant_* | *δ^13^C_diet,i,j_* | Mean value and uncertainty of *δ^13^C_diet,i,j_* is assessed in Equation (11) | Equation (11) |
|  |  |  | The regression between *δ^13^C_diet_* and *δ^13^C_CH4-ruminant_* | The linear regression and confidence interval is estimated considering the uncertainty of  *δ^13^C_diet_* | See Methods and Fig. 2 |

**Supplementary Table 4. Feed commodities allocation into seven major feed categories.** These categories were used in the simple feed model (adapted from ref ^25^).

| **Main feed categories** | **Feed commodities in FAOSTAT^20^** |
| --- | --- |
| Maize | Maize and products |
| Cereals without Maize | Barley and products, Cereals Other, Millet and products, Oats, Rice (Milled Equivalent), Sorghum and products, Wheat and products, Rye and products |
| Oilcrops | Oilcrops Other, Olives (including preserved), Soyabeans, Rape and Mustardseed, Sunflower seed, Soyabean Oil, Cottonseed, Sesame seed, Coconut Oil, Cottonseed Oil, Groundnut Oil, Oilcrops Oil Other, Palm Oil, Palmkernel Oil, Rape and Mustard Oil, Sesameseed Oil, Sunflowerseed Oil, Coconuts - Incl Copra, Palm kernels, Olive Oil, Groundnuts (in Shell Eq), Cocoa Beans and products, Dates, Cotton lint |
| Cakes of Oilcrops | Cottonseed Cake, Oilseed Cakes Other, Sesameseed Cake, Sunflowerseed Cake, Copra Cake, Groundnut Cake, Palmkernel Cake, Rape and Mustard Cake, Soyabean Cake |
| Brans | Brans |
| Pulses | Pulses Other and products, Beans, Cloves, Peas |
| Others | Potatoes and products, Roots & Tuber Dry Equiv, Cassava and products, Roots Other, Sweet potatoes, Yams, Molasses, Sugar beet, Sugar cane, Sugar (Raw Equivalent), Sugar Refined Equiv, Sweeteners Other, Sugar non-centrifugal, Vegetables Other, Apples and products, Fruits Other, Plantains, Bananas, Groundnuts (in Shell Eq), Onions, Tomatoes and products, Tea (including mate), Grapefruit and products, Oranges Mandarines, Grapes and products (excl wine) |

**Supplementary Table 5. Feed commodities allocation into 19 crop products with their dry matter to biomass ratios and carbon to dry matter ratios (adapted from ref ^25^).**

| **Raw product** | **Dry matter to biomass ratio** | **Feed commodities in FAOSTAT**^20^ |
| --- | --- | --- |
| Barley | 0.85 | Barley and products |
| Cotton | 0.85 | Cottonseed Cake, Cottonseed, Cottonseed Oil, Cotton lint |
| Groundnuts | 0.95 | Groundnut Cake, Groundnut Oil, Groundnuts (in Shell Eq), Groundnuts (Shelled Eq) |
| Millet | 0.85 | Millet and products |
| Maize | 0.85 | Maize and products |
| Oilpalm | 0.95 | Palmkernel Cake, Palm Oil, Palmkernel Oil, Palm kernels, Dates |
| Potatoes | 0.25 | Potatoes and products, Roots Other, Sweet potatoes, Yams |
| Pulses | 0.9 | Pulses Other and products, Beans, Peas |
| Rape | 0.85 | Rape and Mustard Cake, Rape and Mustardseed, Rape and Mustard Oil |
| Sunflowers | 0.9 | Sunflowerseed Cake, Sunflower seed, Sunflowerseed Oil |
| Cassava | 0.85 | Cassava and products |
| Rice | 0.88 | Brans, Rice (Milled Equivalent), Rice (Paddy Equivalent) |
| Rye | 0.85 | Rye and products |
| Sorghum | 0.85 | Sorghum and products |
| Soybean | 0.85 | Soyabean Cake, Soyabeans, Soyabean Oil |
| Sugar beets | 0.18 | Molasses, Sugar beet, Roots & Tuber Dry Equiv, Sugar (Raw Equivalent), Sugar Raw Equivalent, Sugar Refined Equiv, Sweeteners Other, Sugar non-centrifugal |
| Sugar cane | 0.14 | Sugar cane |
| Wheat | 0.85 | Wheat and products |
| Others | 0.5 | Oilseed Cakes Other, Sesameseed Cake, Cereals Other, Copra Cake, Oilcrops Other, Olives (including preserved), Vegetables Other, Apples and products, Cloves, Sesame seed, Fruits Other, Coconut Oil, Oilcrops Oil Other, Sesameseed Oil, Plantains, Bananas, Coconuts - Incl Copra, Olive Oil, Cocoa Beans and products, Onions, Tomatoes and products, Tea (including mate), Grapefruit and products, Oranges Mandarines, Grapes and products (excl wine) |

**Supplementary Table 6. Methane sources and their default *δ^13^C* used in the one-box model.**

| Source component | | *δ^13^C* (‰)# | Value and inventory source for 1700 A.D. | Inventory source for 1850-1960 | Inventory source for 1970-2012 |
| --- | --- | --- | --- | --- | --- |
| Natural Sources | |  |  |  |  |
|  | Wetlands | -60 | 163 (Ref ^3^) | Ref ^3^ | Ref ^3^ |
|  | Termites | -57 | 20 (Ref ^3^) | Ref ^3^ | Ref ^3^ |
|  | Oceans | -40 | 15 (Ref ^3^) | Ref ^3^ | Ref ^3^ |
|  | Wild animals | -62 | 15 (Ref ^3^) | Ref ^3^ | Ref ^3^ |
|  | Geologic | -40 | 4 (Ref ^3^) | Ref ^3^ | Ref ^3^ |
| Anthropogenic sources | |  |  |  |  |
|  | Fossil fuel (industry) | -44 | 0 (Ref ^3^) | Ref ^26^ | Ref ^27^ |
|  | Waste treatment and landfill | -55 | 5 (Ref ^3^) | Ref ^26^ | Ref ^27^ |
|  | Rice cultivation | -64 | 10 (Ref ^3^) | Ref ^26,27^ | Ref ^27^ |
|  | Livestock enteric fermentation | -62 | 5 (Ref ^3^) | Ref ^26,27^ | Ref ^27^ |
|  | Manure management | -55 | 0 (Ref ^3^) | Ref ^26,27^ | Ref ^27^ |
|  | Agricultural waste burning | -25 | 0.4 (Ref ^26^) | Ref ^26^ | Ref ^27^ |
|  | Forest burning* | -25 | 4.8 (Ref ^26^) | Ref ^26^ | Ref ^26^ |
|  | C3 grass burning* | -25 | 5.3 (Ref ^26^) | Ref ^26^ | Ref ^26^ |
|  | C4 grass burning* | -12 | 3.9 (Ref ^26^) | Ref ^26^ | Ref ^26^ |

* In this study, we separate four pyrogenic sources: agricultural waste burning, forest burning, C3 grass burning, and savanna (C4 grass) burning. The data of forest burning, C3 grass burning, and savanna (C4 grass) burning in ref ^26^ include both wildfires (a natural source) and anthropogenic burnings. Thus, we do not differentiated wildfires specifically as a natural source.

# For *δ^13^C* of sources other than fossil fuel, the values from ref ^3^ are used. For *δ^13^C* of fossil fuel emissions we did not separate coal mining (-35‰) and other fossil (-40‰) as in ref ^3^. Instead, we used a value of -44‰ for total fossil fuel emissions, which is the time averaged, global weighted mean value from an inventory of ref ^28^. It should also be noted that there are updated *δ^13^C* values for other sources like northern ^29^ and tropical wetlands ^30^. In this study, the purpose of the box-model simulations is to show the impact of our new estimates of livestock emissions on atmospheric trends, which are less sensitive to the default *δ^13^C* values than to the proportions of sources at a given point in time. Thus, we only account for the significant revision of *δ^13^C* of fossil fuel emissions, and the revisions of *δ^13^C* of livestock emissions in this study.

**Supplementary Note 1. An adapted simple feed model used to constrain feedstuffs for poultry, pigs, and ruminants**

Feedstuffs used to feed farm livestock was calculated using a simple feed model^25^ with some adaptation, and the residual feed demand by ruminant livestock was assumed to rely on local forage and grass. The details in the feed model and the methodology were described here and also see ref ^25^.

Six major feeds were considered in original simple feed model from ref ^25^: maize, other cereals, oilseed (oilcrops), oilseed cakes (oilcrops cakes), brans, and pulses. In this study, a category of “others” was differentiated in addition to above six major feed categories (Supplementary Table 4). The “other” category includes mainly starch crops and sugars (ca. 8 – 12% of total dry matter of feed commodities), and few of vegetable and fruit products (ca. 1-2% of total dry matter of feed commodities; Supplementary Table 4). It will be used to first satisfy the energy requirement of pigs. The supply of the major feeds produced on arable areas according to FAOSTAT-Commodity Balances^20^ is distributed to poultry and eggs, pigs and cattle according to nutritional demands as follows: The major feeds encompass maize, other cereals, oilseed cakes/meals, oilseeds, brans, pulses, and others. Other cereals and maize components can be adjusted to available supply.

Poultry receives maize, other cereals and oilseed cakes/meals to satisfy an average demand of 320 MJ head^-1^ year^-1^ and of 5 kg raw protein head^-1^ year^-1^ – a typical value for European poultry production. Pigs receive maize, other cereals, oilseed cakes/meals, brans, pulses, and eventually oilseeds in case the demand is not covered by oilseed cakes/meals. In this study, the “others” category is assumed to supply energy for pigs. A multicomponent mixing model based on the crop properties^31^ determines the feed mix for each country according to the nutritional demand of fattening pigs in Europe (adjusted from ref ^31^: 795 kg dry feed head^-1^ year^-1^, 10328 MJ head^-1^ year^-1^, 138 kg raw protein head^-1^ year^-1^ and 7.3 kg lysine head^-1^ year^-1^, adjusting cereals and maize components to available supply). In developing regions, the feed demand for pigs exceeds the supply available in several cases, so here, we assume that all available feed components are used and then amended by components not considered here. Pulses would be all used for feeding pigs. Pigs would receive oilseeds when cakes of oilseeds are not enough.

**Supplementary References**

1 Herrero, M. et al. Biomass use, production, feed efficiencies, and greenhouse gas emissions from global livestock systems. *Proc. Natl. Acad. Sci. USA.* 110, 20888-20893, doi:10.1073/pnas.1308149110 (2013).

2 Schaefer, H. et al. A 21st-century shift from fossil-fuel to biogenic methane emissions indicated by ^13^CH_4_. *Science* 352, 80-84, doi:10.1126/science.aad2705 (2016).

3 Lassey, K. R., Etheridge, D. M., Lowe, D. C., Smith, A. M. & Ferretti, D. F. Centennial evolution of the atmospheric methane budget: what do the carbon isotopes tell us? *Atmos. Chem. Phys.* 7, 2119-2139, doi:10.5194/acp-7-2119-2007 (2007).

4 González-Martin, I., González-Pérez, C., Hernández Méndez, J., Marqués-Macias, E. & Sanz Poveda, F. Use of isotope analysis to characterize meat from Iberian-breed swine. *Meat Sci.* 52, 437-441, doi:10.1016/S0309-1740(99)00027-3 (1999).

5 Knobbe, N. et al. C and N stable isotope variation in urine and milk of cattle depending on the diet. *Anal. Bioanal. Chem.* 386, 104-108, doi:10.1007/s00216-006-0644-6 (2006).

6 Camin, F., Perini, M., Colombari, G., Bontempo, L. & Versini, G. Influence of dietary composition on the carbon, nitrogen, oxygen and hydrogen stable isotope ratios of milk. *Rapid Commun. Mass Sp.* 22, 1690-1696, doi:10.1002/rcm.3506 (2008).

7 Wilson, G. F., Mackenzie, D. D. S., Brookes, I. M. & Lyon, G. L. Importance of body tissues as sources of nutrients for milk synthesis in the cow, using ^13^C as a marker. *Brit. J. Nutr.* 60, 605-617, doi:10.1079/BJN19880131 (1988).

8 De Smet, S., Balcaen, A., Claeys, E., Boeckx, P. & Van Cleemput, O. Stable carbon isotope analysis of different tissues of beef animals in relation to their diet. *Rapid Commun. Mass Sp.* 18, 1227-1232, doi:10.1002/rcm.1471 (2004).

9 O'Leary, M. H. Carbon Isotopes in Photosynthesis. *BioScience* 38, 328-336, doi:10.2307/1310735 (1988).

10 Cerling, T. E. et al. Global vegetation change through the Miocene/Pliocene boundary. *Nature* 389, 153, doi:10.1038/38229 (1997).

11 Rust, F. Ruminant methane delta (^13^C/^12^C) values: relation to atmospheric methane. *Science* 211, 1044-1046 (1981).

12 Levin, I., Bergamaschi, P., Dörr, H. & Trapp, D. Stable isotopic signature of methane from major sources in Germany. *Chemosphere* 26, 161-177, doi:10.1016/0045-6535(93)90419-6 (1993).

13 Bilek, R. S., Tyler, S. C., Kurihara, M. & Yagi, K. Investigation of cattle methane production and emission over a 24-hour period using measurements of δ^13^C and δD of emitted CH_4_ and rumen water. *J. Geophys. Res.-Atmos.* 106, 15405-15413, doi:10.1029/2001JD900177 (2001).

14 Klevenhusen, F. et al. Efficiency of monolaurin in mitigating ruminal methanogenesis and modifying C-isotope fractionation when incubating diets composed of either C3 or C4 plants in a rumen simulation technique (Rusitec) system. *Brit. J. Nutr.* 102, 1308-1317, doi:10.1017/S0007114509990262 (2009).

15 Klevenhusen, F., Bernasconi, S. M., Kreuzer, M. & Soliva, C. R. Experimental validation of the Intergovernmental Panel on Climate Change default values for ruminant-derived methane and its carbon-isotope signature. *Anim. Prod. Sci.* 50, 159-167, doi:10.1071/AN09112 (2010).

16 Metges, C., Kempe, K. & Schmidt, H.-L. Dependence of the carbon-isotope contents of breath carbon dioxide, milk, serum and rumen fermentation products on the δ^13^C value of food in dairy cows. *Brit. J. Nutr.* 63, 187-196, doi:10.1079/BJN19900106 (1990).

17 Losinger, W. C. Feed-conversion ratio of finisher pigs in the USA. *Prev. Vet. Med.* 36, 287-305 (1998).

18 National Chicken Council. https://www.nationalchickencouncil.org/about-the-industry/statistics/u-s-broiler-performance/. (2018).

19 Rabobank Research. https://research.rabobank.com/far/en/sectors/farm-inputs/Pigs_Might_Fly.html. (2015).

20 FAOSTAT Online Statistical Service (Food and Agriculture Organization (FAO), 2017); http://faostat3.fao.org/.

21 Verheijen, L. A. H. M., Wiersema, D., Hulshoff Pol, L. W. & De Wit, J. Management of Waste from Animal Product Processing, International Agriculture Centre, Wageningen, The Netherlands, January, 1996. Online access: http://www.fao.org/WAIRDOCS/LEAD/X6114E/x6114e04.htm. (1996).

22 Chang, J. et al. Combining livestock production information in a process-based vegetation model to reconstruct the history of grassland management. *Biogeosciences* 13, 3757-3776 (2016).

23 IPCC. 2006 IPCC guidelines for national greenhouse gas inventories. Eggleston H.S., Buendia L., Miwa K., Ngara T. and Tanabe K. (eds). (Institute for Global Environmental Strategies Hayama, Japan, 2006). https://www.ipcc-nggip.iges.or.jp

24 Still, C. J., Berry, J. A., Collatz, G. J. & DeFries, R. S. Global distribution of C-3 and C-4 vegetation: Carbon cycle implications. *Glob. Biogeochem. Cycles* 17, doi:1006.10.1029/2001gb001807 (2003).

25 Ciais, P., Bousquet, P., Freibauer, A. & Naegler, T. Horizontal displacement of carbon associated with agriculture and its impacts on atmospheric CO_2_. *Glob. Biogeochem. Cycles* 21, doi:10.1029/2006gb002741 (2007).

26 Lamarque, J. F. et al. Historical (1850–2000) gridded anthropogenic and biomass burning emissions of reactive gases and aerosols: methodology and application. *Atmos. Chem. Phys.* 10, 7017-7039, doi:10.5194/acp-10-7017-2010 (2010).

27 EDGARv4.3.2. http://edgar.jrc.ec.europa.eu/, last access: September 2018. (2018).

28 Schwietzke, S. et al. Upward revision of global fossil fuel methane emissions based on isotope database. *Nature* 538, 88-91, doi:10.1038/nature19797 (2016).

29 Fisher, R. E. et al. Measurement of the 13C isotopic signature of methane emissions from northern European wetlands. *Glob. Biogeochem. Cycles* 31, 605-623, doi:10.1002/2016gb005504 (2017).

30 Brownlow, R. et al. Isotopic Ratios of Tropical Methane Emissions by Atmospheric Measurement. *Glob. Biogeochem. Cycles* 31, 1408-1419, doi:10.1002/2017gb005689 (2017).

31 KTBL (Kuratorium fur Technic und Bauwessen in der Landwitschaft). KTBL Pocket Book Agriculture 2000/01 (in German), 20th ed., Kuratorium fur Technic und Bauwessen in der Landwitschaft, Munster, Germany. (2000).
